# Comparative Genomics Reveals the Regulatory Complexity of Bifidobacterial Arabinose and Arabino-Oligosaccharide Utilization

**DOI:** 10.3389/fmicb.2018.00776

**Published:** 2018-04-24

**Authors:** Aleksandr A. Arzamasov, Douwe van Sinderen, Dmitry A. Rodionov

**Affiliations:** ^1^A.A. Kharkevich Institute for Information Transmission Problems, Russian Academy of Sciences, Moscow, Russia; ^2^APC Microbiome Institute and School of Microbiology, University College Cork, Cork, Ireland; ^3^Sanford Burnham Prebys Medical Discovery Institute, La Jolla, CA, United States

**Keywords:** bifidobacteria, arabinose, arabino-oligosaccharides, prebiotics, regulatory networks, transcription factors, comparative genomics

## Abstract

Members of the genus *Bifidobacterium* are common inhabitants of the human gastrointestinal tract. Previously it was shown that arabino-oligosaccharides (AOS) might act as prebiotics and stimulate the bifidobacterial growth in the gut. However, despite the rapid accumulation of genomic data, the precise mechanisms by which these sugars are utilized and associated transcription control still remain unclear. In the current study, we used a comparative genomic approach to reconstruct arabinose and AOS utilization pathways in over 40 bacterial species belonging to the *Bifidobacteriaceae* family. The results indicate that the gene repertoire involved in the catabolism of these sugars is highly diverse, and even phylogenetically close species may differ in their utilization capabilities. Using bioinformatics analysis we identified potential DNA-binding motifs and reconstructed putative regulons for the arabinose and AOS utilization genes in the *Bifidobacteriaceae* genomes. Six LacI-family transcriptional factors (named AbfR, AauR, AauU1, AauU2, BauR1 and BauR2) and a TetR-family regulator (XsaR) presumably act as local repressors for AOS utilization genes encoding various α- or β-L-arabinofuranosidases and predicted AOS transporters. The ROK-family regulator AraU and the LacI-family regulator AraQ control adjacent operons encoding putative arabinose transporters and catabolic enzymes, respectively. However, the AraQ regulator is universally present in all *Bifidobacterium* species including those lacking the arabinose catabolic genes *araBDA*, suggesting its control of other genes. Comparative genomic analyses of prospective AraQ-binding sites allowed the reconstruction of AraQ regulons and a proposed binary repression/activation mechanism. The conserved core of reconstructed AraQ regulons in bifidobacteria includes *araBDA*, as well as genes from the central glycolytic and fermentation pathways (*pyk, eno, gap, tkt, tal, galM, ldh*). The current study expands the range of genes involved in bifidobacterial arabinose/AOS utilization and demonstrates considerable variations in associated metabolic pathways and regulons. Detailed comparative and phylogenetic analyses allowed us to hypothesize how the identified reconstructed regulons evolved in bifidobacteria. Our findings may help to improve carbohydrate catabolic phenotype prediction and metabolic modeling, while it may also facilitate rational development of novel prebiotics.

## Introduction

Bifidobacteria are Gram-positive, non-motile, non-spore-forming, anaerobic saccharolytic microorganisms that represent one of the deepest branches within the Actinobacteria phylum (Ventura et al., 2007; Lugli et al., 2017). They are also characterized by relatively high (∼60%) G+C content of their genomes (Lee and O’Sullivan, 2010). The majority of known *Bifidobacterium* species inhabit the gastrointestinal tract of various animals (mostly mammals, though also birds and insects), yet some species may be found in different ecological niches ranging from the oral cavity (Okamoto et al., 2008; Ventura et al., 2009) to fermented milk products (Watanabe et al., 2009).

Since their first isolation from infant feces in 1899 by Henri Tissier (van den Broek et al., 2008; Lee and O’Sullivan, 2010), bifidobacteria have continued to be a subject of study, especially in the last two decades due to increased interest in the functionalities of the GM in (human) health and disease. Particular *Bifidobacterium* species are known to be among the first colonizers of the sterile gastrointestinal tract and predominate in breastfed infants until weaning, partly because of their ability to metabolize human milk oligosaccharides (Favier et al., 2002; Sela et al., 2008; Milani et al., 2017). Despite the fact that their relative contribution to the GM composition decreases up to 2–14% with aging (Odamaki et al., 2016), these microorganisms are considered to be some of the most prevalent and important members of the community. Bifidobacteria are purported to confer several beneficial properties on their host, among others they are believed to elicit anti-carcinogenic, immunostimulatory, and anti-diarrhoeal activities, while they may also prevent intestinal colonization by pathogenic microbiota (Picard et al., 2005; Russell et al., 2011; Arboleya et al., 2016). Due to these claimed beneficial activities, several bifidobacterial species and strains, such as *B. animalis* subsp. *lactis* and *B. longum* subsp. *longum*, are commonly used as probiotics and are included in functional foods (Stanton et al., 2005; Russell et al., 2011).

Another approach to enhance the abundance and activity of bacteria as a beneficial gut commensal is the use of prebiotics. A prebiotic is a non-digestible compound that, through its metabolization by microorganisms in the gut, increases the number of and/or activity of beneficial components of the GM, thus conferring positive physiological health effects on their host (Bindels et al., 2015). Previously it was shown by *in vitro* studies that AOS and their polymers, arabinans, derived from sugar beet can selectivity increase the abundance of bifidobacteria (so-called bifidogenic effect) (Al-Tamimi et al., 2006; Holck et al., 2011; Onumpai et al., 2011; Vigsnæs et al., 2011; Sulek et al., 2014; Moon et al., 2015). However, the desire for a rational design of novel and more effective AOS-based prebiotics is impeded due to the lack of full understanding of catabolic pathways involved and associated regulatory networks.

The sequencing of the first bifidobacterial genome revealed the extensive coding potential to produce a very wide variety of GHs, of which 11 were predicted to be involved in AOS utilization (Schell et al., 2002). Since then efforts have been concentrated on the study of bifidobacterial arabinosidases (predominantly α-L-arabinofuranosidases). Currently, substrate specificity and the mode of action have been described for several of these enzymes in *B. adolescentis* (Van Laere et al., 1997; van den Broek et al., 2005; Lagaert et al., 2010; Suzuki et al., 2013) and *B. longum* subsp. *longum* (Margolles and de los Reyes-Gavilán, 2003; Fujita et al., 2011, 2014; Lee et al., 2011). Despite their functional importance, arabinosidases compose only one step in sugar catabolic pathways. The other parts are arabinose/AOS transporters, none of which have been described in bifidobacteria to this date, and downstream enzymes: L-arabinose isomerase (AraA), ribulokinase (AraB) and L-ribulose-5-phosphate 4-epimerase (AraD). Moreover, the process of utilization of a particular sugar also requires complex regulatory systems in order to coordinate gene expression in response to substrate availability. Carbohydrate utilization pathways are often controlled by DNA-binding transcriptional factors (TFs) that act as repressors or activators and that induce gene expression upon the appearance of a sugar effector (Cohn and Horibata, 1959). A regulon of a given TF is a group of genes and operons controlled by this TF (Rodionov, 2007). Thus, to unveil the complexities of the arabinose and AOS utilization pathways, it is especially important to reconstruct transcriptional networks and elucidate functional parts of a pathway.

In our previous work, we conducted a global analysis of transcriptional regulation of carbohydrate utilization in 10 *Bifidobacterium* genomes by predicting binding sites and reconstructing regulons for 268 TFs (Khoroshkin et al., 2016). The global regulator AraQ was one of the most striking findings since in addition to arabinose utilization genes its regulon contains several genes of central carbohydrate metabolism (Khoroshkin et al., 2016). In the current study, we focused our attention on the arabinose and AOS utilization pathways and associated transcriptional regulation in a broader, non-redundant set of 39 *Bifidobacterium* strains (both of human and non-human origin) as well as in 6 closely related species from different genera of the *Bifidobacteriaceae* family. The main aims were to expand existing regulons, describe new ones and also look at possible evolutionary clues. To tackle these tasks, we implemented a previously described subsystem-based comparative genomic approach which had been successfully used to reconstruct sugar utilization in several lineages of bacteria including *Shewanella* (Rodionov et al., 2010), *Staphylococcus* (Ravcheev et al., 2011), *Bacillus* (Leyn et al., 2013), *Streptococcus* (Ravcheev et al., 2013a), *Bacteroides* (Ravcheev et al., 2013b) and *Thermotoga* (Rodionov et al., 2013). Our results include reconstruction of regulons of 9 orthologous groups of TFs belonging to the LacI, TetR and ROK families. We hypothesize that the majority of these regulators act as local repressors, controlling the expression of co-localized arabinosidases and prospective AOS transporters, whereas AraQ functions as a global regulator, whose mode of action (i.e., acting as a transcriptional repressor or activator) depends on the particular target gene. In summary, the collected data allowed us to build several models of arabinose and AOS utilization in bifidobacteria. Such models in turn facilitated assignment of carbohydrate utilization phenotypes to each analyzed strain and tracing of the possible evolutionary routes of the reconstructed TF regulons.

## Materials and Methods

### Bioinformatics Methods and Databases

The genomic sequences of the analyzed bifidobacteria were obtained from GenBank (Benson et al., 2017). The initial set of available complete and draft genomes of bacterial species from the *Bifidobacteriaceae* family was filtered to exclude closely related strains and species with a 98.65% threshold on 16S rRNA similarity (Kim et al., 2014). We also included two different subspecies of *B. longum* and two strains of *Gardnerella vaginalis* because these closely related strains differed in the presence of the arabinose utilization genes. As a result, we compiled a non-redundant set of 45 genomes representing 38 *Bifidobacterium* species and 5 species of other genera that belong to the *Bifidobacteriaceae* family (Supplementary Table S1). Additionally, we analyzed the orthologous AraQ regulon in three other lineages of Actinobacteria, namely *Arthrobacter arilaitensis, Geodermatophilus obscurus*, and *Stackebrandtia nassauensis*. The whole-genome phylogeny tree for the selected *Bifidobacteriaceae* genomes was build using the Phylogenetic Tree service included in the PATRIC genomic platform (Wattam et al., 2017). Phylogenetic trees for the selected protein families were constructed via the PhyML algorithm (Guindon et al., 2010) in the Seaview software (Gouy et al., 2010), using the default parameters and bootstrap values computed with 100 replicates.

The comparative genome analysis and reconstruction of arabinose and AOS utilization pathways were performed using the subsystems approach (Rodionov et al., 2010) included in the SEED genomic platform (Overbeek et al., 2005). For functional gene annotation and building of the arabinose/AOS catabolic subsystem in SEED we combined existing annotations with information from literature accessed via the PaperBLAST tool (Price and Arkin, 2017) and reference databases including SwissProt for characterized protein annotations (The UniProt Consortium, 2017), KEGG for reference metabolic pathways (Kanehisa et al., 2016), TCDB for transporter classification (Saier et al., 2016) and CAZy for classification of Glycosyde Hydrolases (GHs) (Lombard et al., 2014). Orthologs were identified using BLAST-based protein similarity searches and gene neighborhood analysis implemented in SEED. Cellular localization of GHs was predicted with SignalP (Petersen et al., 2011). The obtained functional roles of proteins were projected across the selected *Bifidobacteriaceae* genomes thus forming the populated arabinose/AOS utilization subsystem (Supplementary Table S3). Based on the identified gene content we assigned each genome with a specific phenotype, representing the predicted ability to metabolize arabinose and different types of AOS.

### Regulon Inference

Reconstruction of TF regulons was performed in two steps. First, we reconstructed arabinose/AOS utilization regulons for five TFs with predicted TF-binding sites (TFBSs) as based on our previous study involving 10 *Bifidobacterium* genomes (Khoroshkin et al., 2016). These TFs include AraQ (example locus tag BL0275); AbfR (BL0543); AauR (BL0185); BauR1 (BL0426) and XsaR (BAD_1207). We then reconstructed regulons for four newly identified TFs with unknown TFBSs that were co-localized on the chromosome with prospective arabinose and AOS utilization genes. These four regulators are AraU (BL0275), AauU1 (BISA_0485), AauU2 (BREU_1998), and BauR2 (BREU_0448). For identification of TFBSs and regulon reconstruction, we used the previously established comparative genomic approach (Rodionov, 2007). Briefly, it includes the following steps: (i) search for a TFBS motif of a given TF; (ii) construction of nucleotide position weight matrices (PWMs) for a discovered (training) set of TFBSs; (iii) genome-wide identification of additional TFBSs in all studied genomes containing a given TF-encoding ortholog utilizing the obtained matrix. For the previously studied five TFs, we implemented PWMs which were built based on information available in the RegPrecise database (Novichkov et al., 2010). For identification of TFBSs for novel regulators, we collected upstream regions of potential TF-regulated genes in each studied genome containing an ortholog of a TF of interest and scanned for potential DNA motifs with palindromic symmetry using the SignalX tool (Mironov et al., 2000). For each identified motif, a PWM was built via the same tool and was subsequently used to search for additional TFBSs and regulon members in GenomeExplorer (Mironov et al., 2000). The search parameters were as follows: (i) a region -350 to +50 bp relative to a predicted start codon; (ii) a TFBS score threshold of 5 for a strong site and 4.75 for weak sites. For the global AraQ regulon, a TFBS score threshold of 4.75 for strong sites and 4.5 for weak sites was employed. To avoid false positive predictions, predicted TFBSs were filtered using the consistency check and phylogenetic footprinting approaches (Rodionov, 2007). Upstream regions of the AraQ-regulated genes were aligned using Pro-coffee (Di Tommaso et al., 2011). Positions of the -10 and -35 promoter elements were determined via PMW-based searches in GenomeExplorer. TFBS motifs were visualized by WebLogo (Crooks et al., 2004). The reconstructed regulons gene content and candidate TFBSs are described in Supplementary Tables S1, S2.

## Results and Discussion

### Regulation of Arabinose Utilization

L-arabinose utilization is defined as a process that includes the transport of this monosaccharide from the extracellular environment into the cell and its subsequent conversion to D-xylulose 5-phosphate via the successive actions of the AraA, AraB, and AraD enzymes (Chang et al., 2015). In our previous bioinformatics analysis of TF regulons in 10 *Bifidobacterium* genomes, we identified the LacI-family transcriptional regulator AraQ that is presumed to control transcription of the *araBDA* operon (Khoroshkin et al., 2016). Using the previously identified PWM for AraQ-binding sites, we checked the conservation of AraQ operators upstream of orthologous *araBDA* genes in a broader set of genomes from the *Bifidobacteriaceae* family. The *araBDA* operon was identified in 36 out of 45 studied genomes, while an *araQ* ortholog was present in the majority of these genomes with a single exception of *Gardnerella vaginalis* ATCC 14019. Search for AraQ-binding sites identified high-scored sites upstream of the *araBDA* operon in each of the above genomes except *G. vaginalis* (Supplementary Table S1). Notably, in three *Bifidobacterium* species, the *araBDA* operon additionally contains the *araE* gene encoding a putative transporter from the Sugar Porter (SP) family, which shares 47% sequence identity with the arabinose-proton symporter AraE from *Bacillus subtilis* (Sá-Nogueira and Ramos, 1997). Co-localization and apparent co-regulation of *araE* with arabinose catabolic genes confirms the proposed by-homology function in arabinose uptake. Interestingly, orthologous *araE* genes were identified in 8 other *Bifidobacterium* genomes, but in those cases, they were not a part of the *araBDA* operon. Candidate AraQ-binding sites were identified upstream of single *araE* genes in 5 of these genomes (Supplementary Table S1). The initial set of 45 *Bifidobacteriaceae* genomes did not reveal any other prospective arabinose transporters that might be regulated by AraQ. In summary, based on our bioinformatics analyses we conclude that AraQ is a conserved regulator of the arabinose catabolism and uptake genes in the *Bifidobactericeae* family.

Additional similarity searches identified *araQ* orthologs linked to the *araBDA* genes in several other families of Actinobacteria including *Micrococcaceae* (representative genome *Arthrobacter arilaitensis*), *Geodermatophilaceae* (*Geodermatophilus obscurus*) and *Glycomycetaceae* (*Stackebrandtia nassauensis*). Although average sequence identity between proteins encoded by these genes and AraQ from bifidobacteria is relatively moderate (∼35%), the identity is considerably higher (up to 90%) within the Helix-Turn-Helix motif involved in DNA binding, suggesting conservation of their AraQ-binding site motifs. The reconstructed AraQ regulons in the above three species include the *araBDA* operon and an additional putative operon, named *araFGH*, encoding a putative sugar transport system from the ABC superfamily (Supplementary Table S1). The identified *araFGH* transporter is located adjacent to the *araBDA* and *araQ* genes in all three genomes. Based on the genomic context and metabolic reconstruction we propose that the novel AraFGH transporter in Actinobacteria is involved in arabinose uptake.

**FIGURE 1:**
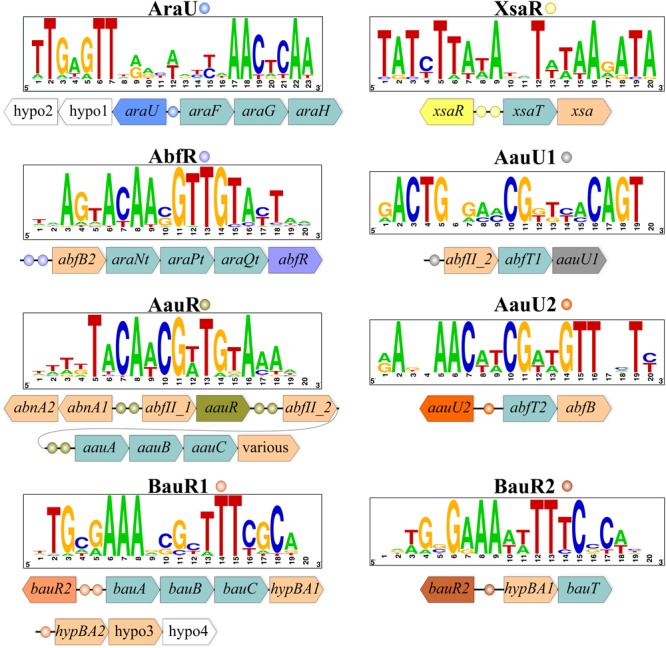
Genomic context and DNA-binding motifs of regulons involved in arabinose/AOS utilization in representative *Bifidobacterium* genomes. For each TF, a consensus DNA binding motif is shown as a sequence logo. TF genes and their associated binding sites are indicated by matching colors. Other genes are shown as arrows colored by their functional classification: arabinosidases, pale brawn; transporters, light green. Genes encoding hypothetical proteins are shown in white. Detailed information about TF regulon distribution and content is given in Supplementary Table S2.

Orthologs of *araFGH* from the above three Actinobacteria were also identified in 19 genomes of the *Bifidobacteriaceae* family. In most of these genomes, *araFGH* is co-localized and divergently transcribed with a gene named *araU*, encoding a putative TF from the ROK family (**Figure 1**). The common upstream regions of the *araU* and *araFGH* genes contain a conserved DNA motif, termed AraU-binding motif (**Figure 1**), which has a similar structure to DNA motifs recognized by other ROK-family regulators (Kazanov et al., 2013). Comparative genomics-based reconstruction of the AraU regulon revealed that the *araFGH* operon was the most conserved regulon member (**Table 1**). Although many previously characterized TFs from the ROK family regulate various sugar utilization pathways (Kazanov et al., 2013), the identified AraU regulator in bifidobacteria is the first example a ROK-family TF that is believed to control arabinose utilization genes.

**Table 1:** Overall information about the distribution of TF involved in arabinose/AOS utilization in 45 studied genomes of the *Bifidobacteriaceae* family and composition of their regulons.

TF regulon	Example locus tag^a^	TF family	Sugar utilized	No.^b^	TF regulon content^c^
AraU	BL1693	ROK	Arabinose	18	*araU* (13), *araFGH* (16)
AbfR	BL0543	LacI	α-AOS	12	*abfR* (12), *abfB2* (12), *araNtPtQt* (6), *afuB-H1* (3), *abfII_2* (1)
AauR	BL0185	LacI	α-AOS	21	*aauR* (17), *abfII_1* (16), *abfII_2* (19), *aauABC* (19), *abnA1* (14), *abnA2* (10), *afuB-H1* (2), *abfBad* (2), *abfC* (1), BDP_0243 (2), BBOMB_ 0938 (1), *abfB2* (1), *araNtPtQt* (1)
AauU1	BISA_0485	LacI	α-AOS	3	*aauU1* (3), *abfII_2* (3), *abfT1* (3)
AauU2	BREU_1998	LacI	α-AOS	3	*aauU2* (3), *abfB* (3), *abfT2* (3)
XsaR	BAD_1207	TetR	α-AOS	5	*xsaR* (5), *xsA* (5), *xsaT* (5)
BauR1	BL0426	LacI	β-AOS	9	*bauR* (9), *hypBA1* (9), *hypBA2* (5), *bauABC* (9)
BauR2	BREU_0448	LacI	β-AOS	5	*bauR2* (4), *hypBA1* (4), *bauT* (3)

*^*a*^Locus tags for each TF gene and genome are given in Supplementary Table S2. ^*b*^Number of genomes possessing a TF. ^*c*^Number of genomes in which a gene is regulated by a TF is given in parenthesis. Distribution of TF regulated genes and TF binding sites is given in Supplementary Table S2.*

Overall, among 36 studied species of the *Bifidobacteriaceae* possessing the arabinose catabolic pathway genes, 29 species contain a potential arabinose transporter (*araE* or *araFGH*) and thus have a metabolic potential to utilize arabinose (Supplementary Table S3). Interestingly, both predicted arabinose transporters are present in two *Bifidobacterium* species, *B. callitrichos* and *B. scardovii*, where *araE* appears to be co-regulated with *araBDA* by AraQ, while *araFGH* is independently controlled by AraU. The presence of two different regulators for arabinose utilization genes in bifidobacteria may be explained by their response to different molecular effectors, e.g., AraU may potentially respond to arabinose, while AraQ may sense a downstream catabolic intermediate such as L-ribulose-5-P. In summary, bifidobacteria demonstrate not only variable arabinose catabolic and transport capabilities, but also appear to employ distinct routes for transcriptional regulation of arabinose utilization genes. This is in contrast to the previously described arabinose regulons in *Escherichia coli* and other enterobacteria (Schleif, 2010), the *Bacillus*/*Clostridium* group (Zhang et al., 2012), *Bacteroides* spp. (Chang et al., 2015), and *Corynebacterium* spp. (Kawaguchi et al., 2009).

### Regulation of AOS Utilization

**FIGURE 2:**
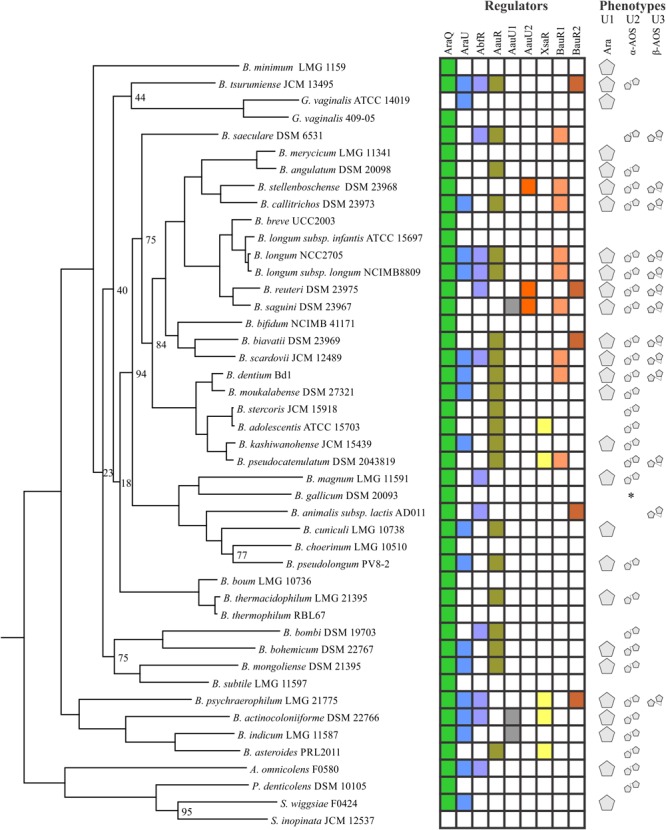
Distribution of arabinose/AOS utilization regulators and predicted growth phenotypes in *Bifidobacteriaceae*. The presence of a particular TF is shown with a colored rectangle. Predicted arabinose, α- and β-AOS utilization phenotypes are schematically represented as pentagons. The special case of arabinose utilization in *B. gallicum* is marked with an asterisk and discussed in the text.

By analogy with arabinose, we defined AOS utilization as a process that includes transporting of an oligosaccharide molecule from the extracellular space into the cell followed by its digestion by various intracellular arabinosidases and the consequent conversion of the released arabinose to D-xylulose 5-phosphate. To identify putative transcriptional regulators of AOS utilization we presumed that local TFs controlling specific sugar catabolic pathway genes are often co-localized with these genes. Consequently, we were able to identify six orthologous groups of TFs that potentially control AOS utilization in bifidobacteria (**Table 1**), and that have a mosaic distribution in 30 out of 45 studied genomes of the *Bifidobactericeae* (**Figure 2**). The identified DNA binding motifs and reconstructed regulons for these local regulators of AOS utilization genes are summarized in **Figure 1** and described in details in Supplementary Table S2. Below we provide the description of these novel TF regulons.

The AbfR regulator from the LacI family was found in 11 *Bifidobacterium* species and *Alloscardovia omnicolens* (**Figure 2**), where it was predicted to control one or two genes encoding intracellular α-L-arabinofuranosidases from the GH51 family, named AbfB2 and AfuB-H1. The latter hydrolase and another closely related paralog from the GH51 family had been experimentally characterized in *B. longum* subsp. *longum* (locus tags BL1166 and BL1611); both of these enzymes recognize and cleave non-reducing terminal α-1,3- and α-1,5- linked L-arabinofuranose (Af) residues (Margolles and de los Reyes-Gavilán, 2003; Lee et al., 2011). Another putative α-L-arabinofuranosidase from the GH43 family (named AbfII_2) belongs to the reconstructed AbfR-controlled regulon, present only in *B. magnum* and *B. longum* strains, while in other bifidobacteria it was predicted to be regulated by another regulator (AauR, see below). Finally, the AbfR-controlled *abfB2* loci, being present in six genomes, contain genes encoding components of a putative carbohydrate ABC transport system, named AraNtPtQt (**Figure 1**). We propose that the AbfR-controlled glycosyl hydrolases and the novel AraNtPtQt transporter are responsible for utilization of α-AOS.

**FIGURE 3:**
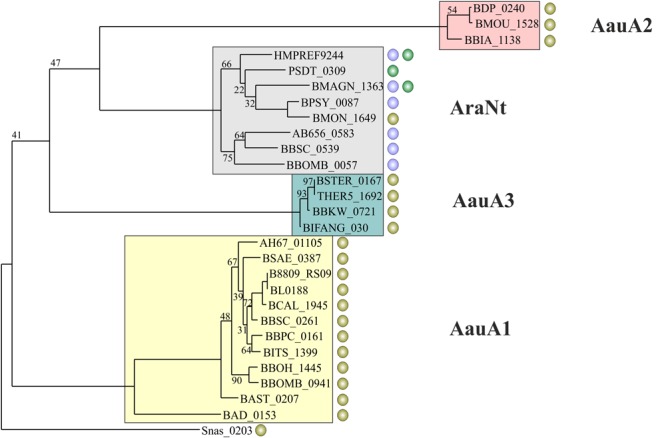
Phylogenetic tree of substrate specific components of ABC transport systems presumably involved in transport of α-AOS. Transporter subunits from the same orthologous group are highlighted with the same background color. The name of each group is given. Circles represent candidate binding sites of various regulators preceding a transporter gene: green – AraQ; purple – AbfR; olive – AauR.

The LacI-family regulator AauR (named for Alpha-Arabino-oligosaccharide Utilization Regulator) is present in 21 *Bifidobacterium* species, making it the most prevalent among other studied TFs after AraQ (**Figure 2**). Composition of the reconstructed AauR regulons from various bifidobacterial species is highly variable. The most conserved regulon members are α-L-arabinofuranosidases from the GH43 family (AbfII_1, AbfII_2), endo-α-1,5-L-arabinanases (AbnA1 and AbnA2) and a putative carbohydrate ABC transport system, named AauABC (**Figure 1** and **Table 1**). The phylogenetic analysis of AauR-regulated ABC transporters suggests that they form three distinct orthologous groups that are unevenly distributed across the *Bifidobacterium* species (**Figure 3**). In several genomes the *aauABC* operons contain additional genes encoding α-L-arabinofuranosidases from the GH1 and GH51 families (termed AbfC and AbfBad, respectively), as well as a putative α-galactosidase and a putative endo-1,4-β-xylanase that are absent in other bifidobacteria (Supplementary Table S2). The intracellular AbfC enzyme (BAD_0156 in *B. adolescentis*) was recently shown to cleave Af from α-1,5-linked AOS in an exo-manner (Suzuki et al., 2013), while AbfBad is a cytoplasmic enzyme that is able to release α-1,3 linked Af residues from double substituted xylose residues in arabinoxylan (Lagaert et al., 2010). The secreted AbnA1 and intracellular AbnA2 arabinases catalyze the internal cleavage of α-AOS and corresponding polysaccharides such as arabinans. The presumably intracellular AbfII enzymes were not experimentally characterized in bifidobacteria; however, their homolog from *Streptomyces avermitilis* (Araf43A, 51% identity) was shown to cleave only terminal α-1,5- linked Af residues of debranched arabinan (Ichinose et al., 2008). The *afuB-H1* and *abfB2-araNtPtQt* genes were found as a part of AauR regulons in several *Bifidobacterium* spp., while in other bifidobacteria these genes belonged to the AbfR regulon. In summary, the AauR regulon core is formed by genes potentially involved in the utilization of linear and branched α-AOS and their polymers, such as arabinans.

Two other groups of LacI-family regulators, named AauU1 and AauU2, control the putative α-AOS utilization operons in certain *Bifidobacterium* species (**Figure 2**). The predicted AauU1 regulon in *B. actinocoloniiforme, B. indicum* and *B. saguini* contains the *abfII_2-abfT1* operon encoding the GH43-family α-L-arabinofuranosidase and a prospective α-AOS permease from the Oligosaccharide:H+ Symporter (OHS) family (**Figure 1**). The *abfT1* permease is an ortholog (49% identity) of the arabinose-induced transporter CAC1530, which is linked to an arabinosidase gene in *Clostridium acetobutylicum* (Zhang et al., 2012). The putative AauU2 regulon in *B. reuteri, B. saguini* and *B. stellenboschense* contains the *abfT2-abfB* operon encoding a cytoplasmic α-L-arabinofuranosidase from the GH51 family and a putative permease from the Drug:H+ Antiporter-1 (DHA1) family (**Figure 1**). We tentatively annotated AbfT1 and AbfT2 as α-AOS transporters that supply substrates for their corresponding α-L-arabinofuranosidases.

Based on our analyses we assigned the BauR1/BauR2 group of TFs to transcriptionally govern genes involved in the utilization of β-AOS that are common in plants and mainly found in various hydroxyproline-rich glycoproteins (Fujita et al., 2014). It was previously proposed that catabolism of β-AOS in *B. longum* subsp. *longum* JCM1217 is controlled by a LacI-family regulator (Fujita et al., 2014). Orthologs of this putative TF (named BauR for Beta-Arabino-oligosaccharide Utilization Regulator) are present in 14 studied *Bifidobacterium* genomes. Detailed analysis of their phylogeny, DNA binding motifs and reconstructed regulons allowed the division of these regulators into two groups, namely BauR1 and BauR2 (**Figures 1, 2**). The BauR1 regulons include the GH121-family β-L-arabinofuranosidase HypBA1, the GH127-family β-L-arabinobiosidase HypBA2, and a prospective β-AOS ABC transport system (named BauABC). The cytoplasmic HypBA1 hydrolase was shown to release β-1,2- linked Af residues from β-arabinobiose (Fujita et al., 2014), while the surface localized HypBA2 enzyme acts on carrot extensin, potato lectin, and various synthetic substrates liberating β-1,2-arabinobiose (Fujita et al., 2011). The reconstructed BauR2 regulons contain orthologs of the cytoplasmic HypBA1 enzyme and a putative transporter from the Glycoside-Pentoside-Hexuronide (GPH) symporter family (named BauT). We propose that the BauR1/BauR2-control glycosyl hydrolases and the novel BauABC and BauT transporters are responsible for utilization of β-AOS.

Finally, the TetR-family regulator XsaR controls the *xsaT-xsa* operon in five *Bifidobacterium* spp. (**Figure 1**). The putative GH43-family α-L-arabinofuranosidase Xsa is similar (36% identity) to exo-α-L-arabinofuranosidase from *B. subtilis*, which was shown to release α-1,2- and α-1,3- linked Af residues from branched arabinan and arabinoxylan (Inácio et al., 2008). A putative DHA1-family permease encoded in the same operon with *xsa* was proposed to be involved in α-AOS uptake. It should be emphasized that XsaR be the first example of a member of the TetR family regulating the process of AOS utilization.

In summary, our bioinformatics analysis of regulons revealed a multitude of regulatory patterns for AOS utilization genes in bifidobacteria (**Figure 2** and Supplementary Table S3). First, the identified AOS-related TFs form 6 orthologous groups that exhibit mosaic distribution among the studied genomes; at that a single strain may possess up to three different AOS utilization regulators. Second, several members of the AbfR, AauR, AauU1, and AraQ regulons, namely *abfB2, abfII_2* and *araNtPtQt*, are either regulated by different TFs in various studied genomes (replaced regulons) or co-regulated by two TFs in some genomes (overlapped regulons). Bioinformatics-mediated reconstruction of arabinose /AOS utilization pathways involving the reconstructed TF regulon members is provided in the following section.

### Reconstruction of Arabinose/AOS Utilization Pathways

The reconstructed TF regulons included a large set of genes that were potentially involved in the utilization of arabinose and AOS (Supplementary Table S3). We further searched for additional genes involved in these metabolic pathways, but that were not found as a part of the reconstructed TF regulons. First, we added orthologs of two additional α-L-arabinofuranosidases from the GH43 family that had been experimentally described in *B. adolescentis*, named AbfA and AXH-D3 (Lagaert et al., 2010). AbfA releases α-1,2- or α-1,3- linked Af residues from monosubstituted xylose residues in arabinoxylan, whereas AXH-D3 releases only α-1,3- linked Af residues from double substituted xylose residues. Orthologs of AbfA and AXH-D3 were found in 21 and 9 studied genomes, respectively (Supplementary Table S3). A putative α-L-arabinofuranosidase from the GH51 family (BL1138 in *B. longum* subsp. *longum*, named AbfA2) was identified in 9 bifidobacteria. The extracellular α-L-arabinofuranosidase AXH-m23 (BL1543) was identified only in two *B. longum* subsp. *longum* strains. A homolog of AXH-m23 in *B. subtilis* (47% sequence identity) was shown to cleave α-1,2- or α-1,3- linked Af from monosubstituted xylose residues (Bourgois et al., 2007). The expression level of BL1543 significantly increased in the presence of arabinoxylan (Savard and Roy, 2009), therefore emphasizing its role in the degradation of this polysaccharide. Finally, two other prospective GH43-family enzymes, AbfE1 (BL0146) and AbfE2 (BL0158), were identified in *B. longum* subsp. *longum* and two other species. These two putative α-L-arabinofuranosidases are characterized by their large size (>950 a.a.) and possible membrane localization. We also predicted that AbfE1 was a part of the expanded AraQ regulon in all four genomes where its ortholog was present (see below).

We further assigned every studied strain with the following predicted phenotypes depending on the presence of genes involved in the transport and catabolism of arabinose and AOS (**Figure 2** and Supplementary Table S3). The U1 phenotype was assigned to 27 strains that were predicted to have a capability to utilize arabinose monosaccharide since they contained both the catabolic genes *araBDA* and the *araE*/*araFGH* transporter genes. The U2 and U3 phenotypes were assigned to 29 and 13 strains that were capable to uptake and catabolize α-AOS and β-AOS, respectively. Ten strains are proposed to possess the ability to metabolize both the arabinose monosaccharide as well as each AOS oligosaccharide type (phenotype U123). In contrast, 10 strains are not able to utilize any of these sugars, including 9 ones with phenotype 0, and a single exception of *B. gallicum* (unique phenotype) that contains the *araBDA* catabolic genes but lacks any known arabinose or AOS transporter (see below).

The analyzed strains demonstrated a mosaic distribution of the predicted arabinose/AOS utilization phenotypes, and even phylogenetically closely related strains might significantly differ in their utilization capabilities (Supplementary Table S3). The most extreme examples were *B. longum* subsp. *longum* and subsp. *infantis*. The strain ATCC 15697 (*B. longum* subsp. *infantis*) lacks the *araBDA* genes, and is thus not expected to utilize any form of arabinose. In contrast, the NCC2705 and NCIMB8809 strains (*B. longum* subsp. *longum*) contain the largest number of genes associated with arabinose/AOS utilization, and are thus assigned the U123 phenotype. In several cases, the related strains were assigned the same phenotype (e.g., U2), yet were shown to possess different α-AOS utilization regulons (e.g., AauU2 in *B. stellenboschense* and AauR in *B. callitrichos*) that included different oligosaccharide uptake systems (AbfT2 and AauABC).

To check the robustness of the obtained metabolic reconstructions, we assessed available experimental data on arabinose and AOS utilization in the *Bifidobacteriaceae*. In the case of arabinose monosaccharide, the predicted phenotypes were in excellent agreement with fermentation data. Thus, we observed concurrence in 20 out of 22 strains, making the accuracy of the prediction of 90% (Supplementary Table S3). All matching cases could be divided into 2 inverse types: (i) when a strain was assigned with the U1 phenotype and had been shown to ferment arabinose, e.g., *B. longum* subsp. *longum* NCC2705; (ii) when a strain had been shown not to utilize arabinose and was assigned with phenotype 0. The only strains with inconsistency between the predicted phenotype and experimental data were *B. pseudocatenulatum* and *Parascardovia denticolens* since they lacked any known arabinose transporter genes (Supplementary Table S3). One possible explanation for this discrepancy may be that the arabinose uptake in these organisms is carried out by a different yet unknown transport system. Unlike arabinose, there are few reports regarding α-AOS utilization by specific strains of bifidobacteria. In one of them it was shown that linear α-AOS, as well as linear arabinan, supports growth of *B. longum* subsp. *longum* ATCC 15707, but not *B. bifidum* ATCC 29521 or *B. longum* subsp. *infantis* ATCC 15697 (Moon et al., 2015). Only the latter strain is included in our reconstruction, and the absence of growth is indeed in agreement with the predicted phenotype (Supplementary Table S3).

### Correlation Between Growth Phenotypes and Strain Isolation Sites

Using published data about isolation sites of the analyzed bifidobacteria we compared them with the predicted abilities of microorganisms to utilize arabinose/AOS (Supplementary Table S3). For instance, strains with the highest utilization capabilities (phenotype U123) were isolated from omnivore animals, such as pigs, marmoset and tamarin monkeys, and humans. These results are in agreement with the previous observation that bifidobacteria of monkey/human origin exhibit a higher number of GH genes in their genomes, which is likely caused by their adaptation to a diversified diet of their hosts (Lugli et al., 2017). Additionally, since the primary source of arabinose/AOS are various plant polysaccharides it is expected that strains isolated from herbivorous organisms would also have rich arabinose/AOS utilization phenotypes. Among the studied bifidobacteria, *B. saeculare* was the only one strain of herbivore origin that was capable of utilizing both arabinose and α- and β-AOS. Two other herbivore-associated strains, *B. magnum* and *B. merycicum*, utilize arabinose/α-AOS and arabinose, respectively. In contrast, *B. boum* could utilize neither arabinose nor AOS.

Among bifidobacteria of human origin, three strains with phenotype 0 (i.e., ones that do not have *araBDA*) were isolated from infants (Supplementary Table S3). This observation is in line with the fact that the majority of such strains predominate in the infant’s gut due to their specialization on utilizing non-digestible human milk oligosaccharides (Sela et al., 2008; Pokusaeva et al., 2011). The ability of 5 other infant-derived strains to somehow utilize arabinose or/and AOS, might reflect their niche-specific adaptation, i.e., specialization to consuming of sugars other than HMO to avoid competition. One may speculate that with growing of the host and change of the diet, only *Bifidobacterium* strains that can utilize plant-derived sugars such as AOS can survive. The transition to arabinose/AOS utilization may also be dictated by other members of GM. This notion is based on the discrepancy between the number of intracellular and secreted arabinosidases, and the dearth of latter ones suggests that bifidobacteria rely on other bacteria that can digest polysaccharides to oligosaccharides (Supplementary Table S3). In summary, it would be interesting to check whether utilization phenotypes are to a higher extent associated with the diet rather than with the taxonomy of the host; however, additional data such as diet composition of hosts from which these bacteria were isolated are needed to check this hypothesis.

### Models of Arabinose and AOS Utilization in Bifidobacteria

**FIGURE 4:**
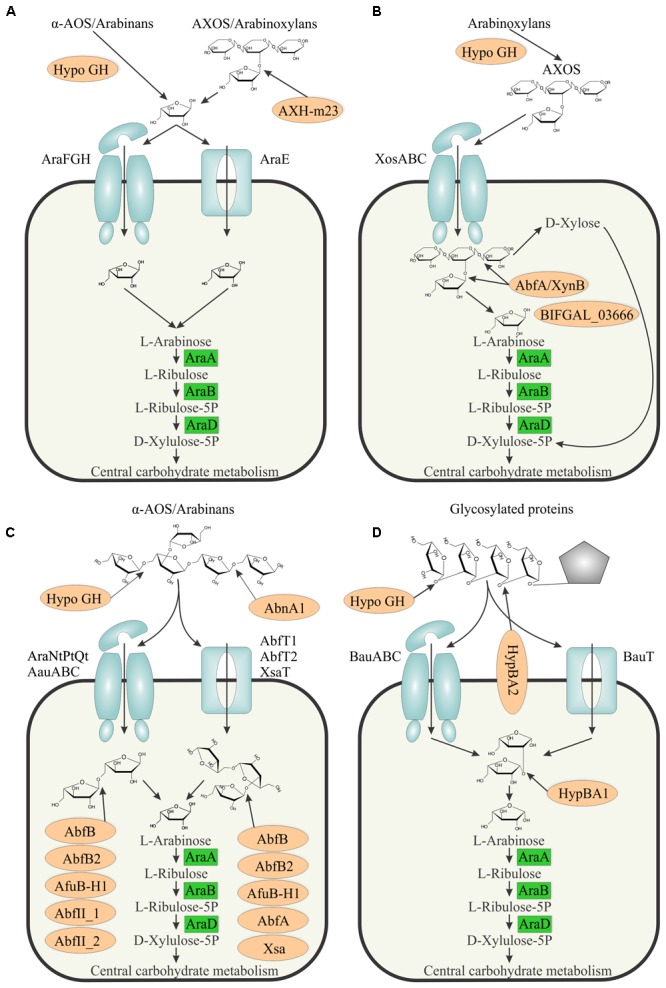
Models of arabinose and AOS utilization in bifidobacteria. **(A)** Arabinose utilization via arabinose transporters. **(B)** Arabinose utilization in *B. gallicum*. **(C)** α-AOS utilization. **(D)** β-AOS utilization. Arabinosidases are shown by light brown ovals. Predicted sugar uptake ABC transporters and permeases are shown as light blue complex and rectangular shapes, respectively. The arabinose monosaccharide catabolic enzymes are shown in green boxes. ‘Hypo GH’ denotes hypothetical glycosyl hydrolases that are presumed to be involved in AOS utilization. Pentagon represents a hydroxyproline residue of a glycosylated protein.

Based on the inferred reconstructions we proposed the following models of arabinose and AOS utilization in bifidobacteria (**Figure 4**). In each of these models, the intracellular arabinose is converted to D-xylulose-5P via the committed catabolic pathway involving the AraA isomerase, AraB kinase, and AraD epimerase enzymes. The latter product is further metabolized through the bifid shunt (Pokusaeva et al., 2011). The obtained metabolic reconstruction suggests three major models and one additional scenario for utilization of arabinose from various exogenous sources in bifidobacteria.

In the first model (**Figure 4A**), arabinose is transported into the cell via AraE (permease) or AraFGH (ABC-type) transporters. The release of terminal arabinose residues (mainly Af) of various oligosaccharides such as AOS and AXOS is performed by various extracellular GH (mainly α-L-arabinofuranosidases). Our genomic analysis suggests that only two strains of *B. longum* subsp. *longum* contain the extracellular arabinofuranosidase AXH-m23, which is presumably implicated in the AXOS utilization. In confirmation, *B. longum* subsp. *longum* NCC2705 was shown to consume only arabinose substituents of AXOS and not the xylose backbone (Rivière et al., 2014, 2015). AbfE1 and AbfE2 are two other candidates for arabinose-cleaving activity outside the cell due to their predicted membrane localization. Remarkably, these two enzymes were also rare among the studied species and were found in *B. longum* subsp. *longum* and 3 other species of bifidobacteria (Supplementary Table S3). These observations indicate that only a few *Bifidobacterium* species are capable of cleaving and utilizing extracellular arabinose, while the majority of them utilize free arabinose that is released from oligo- and polysaccharides by other microbial community members. The suitable candidates for such reliance in GM are the *Bacteroides* species due to a significant diversity of their arsenal of secreted GH (Ravcheev et al., 2013b).

In the second model (**Figure 4C**), branched and debranched arabinan and long α-AOS molecules are degraded by extracellular endo-α-1,5-L-arabinanases, followed by cellular uptake of the generated α-AOS by means of ABC- or permease-type uptake systems, after which the internalized oligosaccharides are hydrolyzed to arabinose using a variety of intracellular arabinofuranosidases targeting terminal α-1,5-, α-1,2-, and α-1,3- bound Af residues. Using regulon analysis, we identified seven groups of putative α-AOS transporters, which included AraNtPtQt, three groups of AauABC ABC transport systems and three permease-type systems, AbfT1, AbfT2 and XsaT (Supplementary Table S3). Genome context analysis in combination with available data on the specificity of the arabinofuranosidases allowed us to assign the most likely substrates of these transporters. Thus, we deducted that AauABC(1-3) and AbfT1 presumably transported linear α-AOS based on their co-occurrence and co-regulation with α-L-arabinofuranosidases releasing α-1,5-linked Af (AbfII_1, AbfII_2, AbfC). However, the AauABC(3) system may also transport branched α-AOS, since in some species genes of this transporter are coupled with AbfBad, which releases only α-1,3- linked Af residues. For AraNtPtQt and AbfT2, we predict uptake of both branched and linear α-AOS based on their genomic coupling with AbfB2 and AbfB, respectively. Finally, XsaT presumably transports only branched α-AOS. Diversification of α-AOS transporters based on substrate length, a degree of branching and modifications such as feruloylation (Holck et al., 2011) and their mosaic distribution among species of bifidobacteria imply possible co-degradation of AOS by various species that alleviates competition through substrate specialization.

The third model describes utilization of β-AOS released from glycosylated proteins (**Figure 4D**). The first step is the release of the terminal Af residue from a β-AOS chain bound to a hydroxyproline residue, followed by the release of β-arabinobiose. In *B. longum* subsp. *longum*, it was shown experimentally that the second step of this process is carried by the membrane-bound β-L-arabinobiosidase HypBA2, while the first step was predicted to be performed by a hypothetical GH encoded by BLLJ_0213, which is located in the same operon with *hypBA2* (Fujita et al., 2014). The liberated disaccharides are transported into the cell via one of the two predicted transport systems, BauT (a permease) and BauABC (an ABC transporter). Intracellular β-arabinobiose is further hydrolyzed by HypBA1, thus producing two arabinose residues (Fujita et al., 2014). Since *hypBA2* is missing in several *Bifidobacterium* strains possessing other β-AOS utilization genes (Supplementary Table S3), we speculate that other unidentified GHases may be involved in these extracytoplasmic reactions. Alternatively, these strains may depend on enzymatic capabilities of other GM species for release of and cross-feed on β-AOS disaccharides.

An additional scenario for utilization of arabinose released from degradation of AXOS was proposed for *B. gallicum* (**Figure 4B**). This strain is unable to utilize arabinose from arabinose/xylose mixture (Rivière et al., 2014), which is in agreement with the absence of any predicted arabinose transporter genes in its genome (Supplementary Table S3). *B. gallicum* is the only studied species whose genome contains the *araBDA* operon yet does not specify any known pathway for AOS utilization (**Figure 2**). We hypothesize that this strain not only internalizes xylooligosaccharides (XOS) but also AXOS via the prospective transporter XosABC (BIFGAL_03672-3669-3667). The AXOS are then further broken down by AbfA/XynB (BIFGAL_03676) and a GH43-family enzyme (BIFGAL_03666). This alternative strategy of arabinose utilization may also be operational in other species possessing *araBDA, xosABC*, and *abfA/xynB* orthologs.

### Global AraQ Regulon

Our results suggest that AraQ is a master regulator of the arabinose catabolic pathway genes *araBDA* and the arabinose transporter gene *araE* in the *Bifidobacteraceae* family. However, AraQ was also found in seven *Bifidobacterium* strains (e.g., *B. breve* UCC2003 and *B. longum* subsp. *infantis* ATCC 15697) and a single strain of *G. vaginalis* that lacked the arabinose utilization genes, suggesting it is involved in transcriptional control of other metabolic pathways. *Scardovia inopinata* and another strain of *G. vaginalis* (ATCC 14019) were the only two strains from the *Bifidobacteraceae* family that lacked an *araQ* ortholog (**Figure 2**). The comparative genomics reconstruction of orthologous AraQ regulons allowed identification of the conserved core regulon genes and genome-specific regulated genes in the studied 43 genomes of *Bifidobacteraceae* (**Table 2** and Supplementary Table S1). The core regulon members were defined as genes preceeded by potential AraQ-binding sites that were conserved in 20 or more genomes. The core regulon includes the *araBDA* and *araQ* genes and seven genes involved in the central carbohydrate metabolism (*gap, eno, pyk, tkt-tal, galM*) and lactate fermentation (*ldh*). At that, the pyruvate kinase *pyk* belongs to the AraQ regulon in all studied *Bifidobacterium* genomes and in *G. vaginalis*, while the enolase *eno* appears to be under AraQ control in 35 genomes.

**Table 2:** Composition of the AraQ regulons in 45 studied genomes of the *Bifidobacteriaceace* family.

Gene/operon	Functional role(s)	No. ^a^	Mode ^b^
*araQ*	Global transcriptional regulator AraQ	20	R
*araBDA*	Ribulokinase; L-ribulose-5-phosphate 4-epimerase; L-arabinose isomerase	35	R
*araE*	Arabinose-proton symporter	8	R
*abfE1*	α-L-arabinofuranosidase AbfE1, GH43	4	ND
*abfB2*	α-L-arabinofuranosidase AbfB2, GH51	3	ND
*araNtPtQt*	α-AOS ABC transport system	3	ND
*gap*	Glyceraldehyde 3-phosphate dehydrogenase	24	A
*eno*	Enolase	35	A
*pyk*	Pyruvate kinase	40	A
*tkt – tal*	Transketolase; transaldolase	24	A
*ldh*	Lactate dehydrogenase	20	A
*galM*	Galactose mutarotase	22	R
*pta – ackA*	Phosphotransacetylase; acetate kinase	8	R
*pflB – pflA*	Pyruvate formate lyase	10	A
BL1113	Aldo/keto reductase	6	R
*malE*	Maltose-binding periplasmic protein	9	A
*malQ1*	4-α-glucanotransferase	4	A?
*maa*	Maltose *O*-acetyltransferase	7	R
*fucO*	Lactaldehyde reductase	7	ND
*glgB –* Blon_ 1762*–*1763	1,4-α-glucan branching enzyme GlgB; two-component regulatory system	6	R
*carD – ispF*	CarD transcriptional regulator; *2-C*-methyl-D-erythritol 2,4-cyclodiphosphate synthase	6	R
*birA*	Biotin-(acetyl-CoA-carboxylase) ligase	6	R
*sixA*	Phosphohistidine phosphatase	5	R
*glk*	Glucokinase, ROK family	7	A
*bfrBCD – X – bfrR – bfrA*	Fructosides ABC transport system; predicted transcriptional regulator; β-fructosidase	4	A

*^*a*^Number of genomes in which a gene is regulated by AraQ. Detailed distribution of AraQ-regulated genes and AraQ-binding sites is given in Supplementary Table S1. ^*b*^Predicted mode of AraQ-dependent regulation: A, activation; R, repression; ND, not determined.*

The less conserved members of the AraQ regulon include the acetate and formate fermentation genes (*pta-ackA* and *pflB-pflA*), a lactaldehyde reductase (*fucO*), the arabinose transporter (*araE*), various arabinofuranosidases and arabinoside transporters, a glucokinase, an aldo/keto reductase and the fructosides catabolism operon *bfr*. Other putative members of the AraQ regulon such as *malE, glgB, carD-ispF* are conserved in a narrow taxonomic group of bifidobacteria including *B. longum* and *B. breve*, while *maa* is preceded by AraQ sites predominantly in the *B. dentium* group. It may be speculated that the latter cases illustrate relatively recent genome- or group-specific regulon expansions.

**FIGURE 5:**
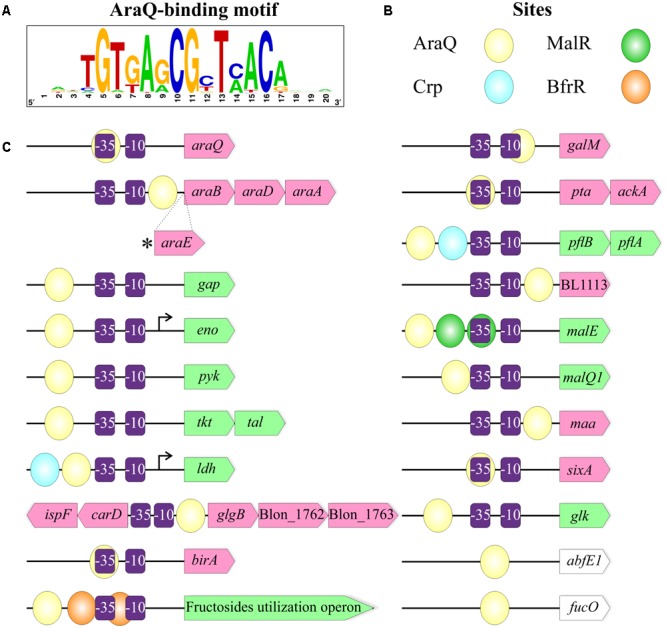
The overall content of the AraQ regulons in bifidobacteria. **(A)** Consensus DNA motif for AraQ-binding sites represented as a sequence logo build based on all identified sites in 45 studied genomes. **(B)** A scheme representing the coloring of different TF-binding sites. Each sphere corresponds to a specific TFBS. **(C)** The overall content of the AraQ regulon. The predicted mechanism of the AraQ action on the expression of a gene of interest is shown by specific coloring: repressed genes are in pink, activated – in green, the ones for which the mode of action was not determined – in white. Prospective –10 and –35 sequences of a bacterial promoter are shown as purple rectangles. Experimentally described transcription start sites are indicated by arrows. The picture represents maximum content of the AraQ regulon, detailed information about its composition in every strain can be found in Supplementary Table S1.

The observed diversity of functional roles and pathways constituting the reconstructed AraQ regulons suggests that AraQ potentially functions as a dual regulator for various genes. To predict the activation or repression mechanism of AraQ action, we investigated multiple alignments of upstream gene regions containing AraQ-binding sites and predicted the conserved -35 and -10 promoter elements (Supplementary Figure S1). The negative mode of regulation was proposed for genes preceded by an AraQ site, which was either located downstream of the -10 element or if it overlapped with the -10/-35 elements. The positive mode of regulation was assumed for AraQ sites that were situated upstream of the -35 element. The overall results of this analysis are presented in **Table 2** and **Figure 5**. The repression mechanism of AraQ action was proposed for the arabinose utilization genes *araBDA*, the regulatory gene *araQ*, the acetate fermentation operon *pta-ackA*, and other genome-specific regulon members including the *galM*, BL1113, *maa, sixA, birA* and *glgB* genes. In contrast, the activation mechanism of AraQ action was proposed for the majority of core regulon genes involved in the central carbohydrate metabolism (*gap, eno, pyk, tkt*-*tal, ldh*), as well as for the maltose and glucose utilization genes (*malE, malQ1, glk*) and the formate fermentation operon *pflBA*. The predicted promoters of two genes, namely *eno* in *B. bifidum* and *ldh* in *B. longum*, were validated using data on experimentally mapped transcription start sites (Minowa et al., 1989; Sun et al., 2014). The obtained results suggest that AraQ may function both as a repressor and an activator of different sets of genes involved in the central carbohydrate metabolism and fermentation.

We also observed that some of the AraQ-regulated genes were potentially controlled by additional TFs (**Figure 5**). The upstream regions of *ldh* and *pflB-pflA* also contain putative binding sites of Crp, another prospective global regulator in bifidobacteria (Khoroshkin et al., 2016). The upstream positions of both of these operator sites relative to the -35 promoter element suggest that both AraQ and Crp might act as transcriptional activators for these fermentation genes. The *malE* gene is additionally controlled by the potential maltose repressor MalR, while the *bfr* operon is co-regulated by the putative beta-fructoside repressor BfrR (Khoroshkin et al., 2016) (Supplementary Figure S1).

### Evolutionary Scenario of the Reconstructed Regulons

Analysis of TF distribution and tentative reconstruction of the regulatory networks allowed us to propose a hypothesis about the possible evolution of the arabinose and AOS regulons in the *Bifidobacteriaceae* genomes. The global AraQ regulon containing central carbohydrate metabolism genes in addition to the arabinose utilization genes was identified in the majority of the *Bifidobacterium* species (Supplementary Table S1). *G. vaginalis* is closely related to the *Bifidobacterium* species according to the whole-genome phylogeny tree (**Figure 2**) and the previous phylogenomic analysis of the *Bifidobacteriaceae* family (Lugli et al., 2017). Among two analyzed strains of *G. vaginalis*, the AraQ regulon in the 409-05 strain contains the central carbohydrate metabolism genes, whereas the AraQ regulator is missing from another strain, ATCC 14019, likely due to a recent gene loss. In contrast, the local AraQ regulons containing only genes involved in arabinose utilization were identified in three other species from the *Bifidobacteriaceae* family (*A. omnicolens, P. denticolens, Scardovia wiggsiae*), as well in *Arthrobacter arilaitensis, Geodermatophilus obscurus*, and *Stackebrandtia nassauensis* representing different taxa of Actinobacteria. These observations lead to the hypothesis that the last universal common ancestor of the *Bifidobacteriaceae* family used AraQ for the control of arabinose utilization genes *araBDA*, whereas the AraU regulator controlled the arabinose ABC transporter genes *araFGH*. In the common ancestor of *Bifidobacterium/Gardenella*, the AraQ regulon expanded to include the central carbohydrate metabolism genes, at that some species lost the arabinose utilization genes but retained AraQ. In some bifidobacteria, e.g., in the *B. longum* subsp. *longum* and subsp. *infantis*, the regulon expansion went to the extreme extent, increasing its size up to approximately 20 genes. This is the first report of regulon expansion in bifidobacteria, although lineage-specific expansions of other central carbohydrate metabolism regulons were documented for other microorganisms, for example, HexR in *Shewanella* (Leyn et al., 2011) and FruR in Enterobacteria (Ravcheev et al., 2014).

The majority of the identified AOS utilization regulators have a narrow taxonomic distribution being present only in the *Bifidobacterium* genus (**Figure 2** and Supplementary Table S2). However, the AbfR regulon was also found in *A. omnicolens*, suggesting its appearance in the common ancestor of *Bifidobacterium* and *Alloscardovia* genera. Interestingly, the AauU2 regulon was only found in species isolated from tamarins and marmoset monkeys, suggesting its late taxon-specific appearance. The AbfR and AraQ regulons show several interconnections including the cases of mosaic regulation and potential co-regulations (Supplementary Table S3). First, the α-AOS utilization genes in *P. denticolens* are controlled by AraQ, whereas in members of the *Bifidobacterium* genus their regulation is intercepted by AbfR. Second, the α-AOS utilization operons in *A. omnicolens* and *B. magnum* are preceded by both AraQ- and AbfR-binding sites suggesting both regulators are involved in the simultaneous control of these genes. Finally, the variable composition of AauR regulons in the *Bifidobacterium* genus was likely shaped by numerous horizontal gene transfer and regulon expansion events. Overall, we believe that the emergence of AOS utilization regulons in bifidobacteria reflects adaptation of these species to the gastrointestinal tract and the lifestyle of feeding on non-digestible plant sugars that are derived from the diet of their host.

## Conclusion and Future Perspectives

This study significantly broadens our understanding of arabinose and AOS metabolism and its transcriptional regulation in bifidobacteria. It was shown in previous works that (i) many members of the *Bifidobacterium* genus are able to ferment arabinose; and (ii) AOS and their polymers of plant origin are causing the bifidogenic effect *in vitro* and therefore can potentially be used as prebiotics. However, a knowledge gap exists with respect to arabinose/AOS utilization and associated transcriptional regulation in bifidobacteria. In the current work, we used comparative genomics approaches to reconstruct metabolic pathways and transcriptional regulons for arabinose and AOS utilization in 39 *Bifidobacterium* strains as well as in 6 strains of closely related genera. Overall, our analysis revealed a complex regulatory network operated by 9 TFs from LacI, TetR and ROK families (**Figure 1** and **Table 1**). The reconstructed TF regulons allowed us to predict several prospective transporters for arabinose and α- and β-AOS. As a result, we describe here a comprehensive collection of genes encoding arabinose/AOS catabolic enzymes and transporters (Supplementary Table S3), predicted sugar utilization phenotypes for each studied strain (**Figure 2**), and propose models for arabinose/AOS utilization in bifidobacteria (**Figure 4**). The observed diversity of arabinose/AOS utilization pathways can be explained by an adaptation of bifidobacteria to survive in the competitive gut environment via their specialization to grow on different sugars (e.g., different AOS). Our results also suggest that the majority of bifidobacteria rely on the ability of other GM bacteria to break down the complex arabinose-containing polysaccharides such as arabinans. The inferred arabinose/AOS utilization phenotypes bring us closer to the ultimate goal of the rational design of AOS-based prebiotics that might positively affect human health and alleviate diseases. This notion is supported by several publications indeed reporting that these sugars are not digested by human secretions (Suzuki et al., 2004; Moon et al., 2015). However, it should be pointed out that the process of prebiotic development should consider metabolic connections of bifidobacteria with other members of the GM, for example if they are dependent on GH activities produced by other bacteria in order to gain access to certain carbohydrates.

Unlike other investigated TFs AraQ is conserved in all studied members of the *Bifidobacterium* genus including species with missing *araBDA* operon. The inferred AraQ regulon suggests that AraQ acts as a global regulator of the central carbohydrate metabolism genes in bifidobacteria. Comparative analysis of AraQ-regulated promoter regions among *Bifidobacterium* strains suggests that AraQ can potentially act as a bi-functional regulator, which represses the *araBDA* and several other genes, while it activates many other genes involved in the central glycolytic pathways (**Figure 5**). The obtained bioinformatics predictions are sufficiently interesting to warrant further experimental verification of AraQ-dependent regulation using both *in vitro* and *in vivo* techniques. Other novel regulators of arabinose and AOS utilization predicted for the first time in this work also await experimental validations, as well as testing of the ability of different *Bifidobacterium* strains to utilize various AOS to confirm predicted utilization phenotypes.

## Author Contributions

AA and DR conceived and designed the research project. AA performed the comparative genomic analysis, reconstruction of TF regulons and building of models of arabinose and AOS utilization. DR provided the quality control of the obtained data. AA, DR, and DS wrote the manuscript. All authors read and approved the final manuscript.

## Conflict of Interest Statement

The authors declare that the research was conducted in the absence of any commercial or financial relationships that could be construed as a potential conflict of interest.

## Abbreviations

Af: arabinofuranose
AOS: arabino-oligosaccharides
AXOS: arabino-xylooligosaccharides
GH: glycoside hydrolase
GM: gut microbiota
PWM: position weight matrix
TF: transcription factor
TFBS: transcription factor-binding site
