# Supplementary material for: Comparative Genomics Reveals the Regulatory Complexity of Bifidobacterial Arabinose and Arabino-Oligosaccharide Utilization
Source: Front Microbiol. 2018 Apr 24;9:776. doi: 10.3389/fmicb.2018.00776 (PMC5928203; doi:10.3389/fmicb.2018.00776)
Supplement: Supplementary file 4 [file Data_Sheet_1.PDF]

FIGURE S1. Multiple sequence alignments of gene upstream regions containing candidate AraQ-binding sites in *Bifidobacterium* spp. Predicted AraQ-binding sites are highlighted in mild yellow (when several overlapping sites are present the weaker one is marked with a rectangle box). Candidate binding sites for Crp, MalR and BfrR1 are in blue, green and orange, respectively. Predicted promoter elements (-35 and -10 boxes) and shown in bold purple. Known transcription start sites are marked in bold red. Putative ribosome binding sites are shown in bold dark red. Coding sequences are in bold, initiation codons are underlined. Conservative nucleotides are marked with asterisks.

1. *araQ* (repression)

```
BDP_1926     TGCGGCTCGACTTGCCCGTCTGGCAGCTCG--GTAAGTAAGTTCGCCGGTGGTTGCCGGCATGCACGAATGCTGCACTAAGTGAACGCGAGAGAACGCCTGTTAGCGCTAACTGAAGTTGCGTGTAGAATGGTCGAAG
BMOU_0432    CGCGGCTCGGCTTGCCCGTCTGGCGGCTGAATGAGCGTAGCCGTCCCAGCGGCGACCGACACATGCGGATACTGCGGCAAACAGGCG-GAGAGAACGCCTGTTAGCGCTAACTGAAGTTGCGTGTAGAATGGTCGGAG
BBPC_1565    CGCTGCGCGACTCGCTCAAATGGCT------------------------------------------GAAAAT--------------------AGCGTCTGTTAGCGCTAACCGAAGTTGCGTGTAGAATAGTCGAAA
BBKW_1660    CGCAGCACGACTTGCTCAAATGGCT------------------------------------------GAAAAT--------------------AGCGTCTGTTAGCGCTAACCGAGGCTGCGTGTAGAATAGTCGAAA
BAD_1413     CGCGGCGCGTCTGCGTGCTTTGGCGGCC----GTGCGGTAGCCTGCGCGCAGTGTGCCGCATCCTTGGAGCAT--------------------GAGGGTTGTTAGCGCTAACTGAAGTTGCGTGTACAATAGGCGAAC
BSTER_1634   CGCGGCGCGTCTGCGTGCTTTGGCGGCC----GTGCGGTAGCCTGCGCGCAGTGTGCCGCATCCTTGGAACAT--------------------GACGGCTGTTAGCGCTAACTGAAGTTGCGTGTACAATAGGCGAAC
              ** ** ** **        ****                                          * *   *                       *  ************* ** * ******** *** * ** *

BDP_1926     GCAACAATGGTCGCGTGTGGAGGGCAATGAGGCATGGCAGTCAGGCGTAAAACGGCGAACAAGCGTCCTTCCATGTTCGAGGTCGCCAAGCTTGCCGGAGTG
BMOU_0432    GCAACGACGGTCGTGTGTGGAGGGCGATGAGGCATGGCAATCAAACGGAAAACGGCGAATAAGCGTCCTTCCATGTTCGAAGTCGCCAAGCTCGCCGGGGTG
BBPC_1565    GTAACAACGGTAGTGTGCGGAAGGCGACGGAGCATGGCAGGCAAGCGCAATGCGGCAAACAAACGCCCCTCGATGTTCGAAGTGGCGAAAATGGCTGGTGTC
BBKW_1660    GTAACAACGGTAGTGTGCGGGAGGCGACGGGGCATGGCAGGCAGGCGTAATGCAGGCAAAAAACGTCCTTCGATGTTCGAAGTGGCAAAATTCGCGGGAGTG
BAD_1413     GTACCAACAGTTGTGTGCGAAAGGCGACGGGGCATGGCAGGCAAACGCGATGCGGGCAATAAGCGTCCTTCGATGTTCGAAGTGGCGAAGCTGGCTGGTGTG
BSTER_1634   GTACCAACAGTTGTGTGCGAAAGGCGACGGGGCATGGCAGGCAAACGCAATGCGGGCAATAAGCGTCCTTCGATGTTCGAAGTGGCGAAGCTGGCTGGTGTG
             * * * *  ** * *** *   *** * *  ********  **  **  *  * *  ** ** ** ** ** ******** ** ** **  * ** ** **
```

2. *araB - araD - araA* (repression)

```
                                                                                     AraQ site 2   AraQ site 1
BSTEL_2135      -------TCAAACCGTTATCT-TTCAGGGCACGTCGGGGTTGTGATACGGCGCTGGGGCCGTTACAATGAATA-CGGAAATTGTGAGCGTTAACGACCACCCAGGGAAGGGGTGAGGT-CAGCGCCCAAGGAACG
BCAL_0892       -------TCAAACCGTTATCG-GACAAGACACGACGAGGTTGAGTTCCATCGGTCCAGTCTGTACAATGGAGCACGGAAATTGTGAGCGTTAACAGCCTGCCCGATGAGGGGCTGGAC-CAACGCCCGCAGGACG
BREU_1992       -------TCGAACCGTTATCTACGACACAACGCTATAAGTTGTGTTGAACCCCAGTTATCGGTACATTGTTTTCTGGAAATTGTGAGCGCTAACAAAAACCCCAAGGAAGGGGAAGGA-ACCCCTTGGT---G--
BISA_1061       -------TCGATTCGTTATAAACGAAATAACGCTATAAGTTGTGTCGAACCTCGGTTATAGGTACAATAATTTTCGGAAATTGTGAGCGCTAACAGAAGTGCCCCAAAGAAGGGGAGC-AAACCCCGGC---G--
BL0274          -------TCAAACCGTTATGATGCGTGACACGGCATAAGTTGTGTCGAATGCGGGTTGTCGGTACAATGTTTTTTGGAAATTGTGAGCGCTAACACCCTCCCCAAGGAAGGGGATAAC-AACGCCGGC-------
B8809_RS01645   -------TCAAACCGTTATGATGCGTGACACGGCATAAGTTGTGTCGAATGCGGGTTGTCGGTACAATGTTTTTTGGAAATTGTGAGCGCTAACACCCTCCCCAAGGAAGGGGATAAC-AACGCCGGC-------
BBIA_1211       -------TAT-CCCGCTATCAATTCGAGACACGTCGAGCTTGTGCTCATCCACTGACCTCGCTACAATGAA--ACCGTTGATGTGAGCGCTAACAGTTCTCCGAAGGAGTAGATGCGG-CGCTTGGGA-------
BBSC_2142       -------TATGAAGGTTATGAAGATGTGACACGGCGAAGTTGTGAAATCCTGCCGTTCCCGCTACAATGAGAACG--TAAATGTGAGCGCTAACAACCCTCCAACGAGGCAGGTGCGA-ACGCTCAAG---GGCA
BDP_1924        AATCAGGCGAATTGCATGGAGTGGCAGGACACGGCGGACTTGTGTGGTCGATGCTTTCCGTTTAGAATAGTGAGCGTTAACAGTGAGCGCTAACATTCGGTTGACAGAGTGGTTACG-AGGCGCTCGG-------
BMOU_0433       -------TCGCCTTGTGGGAACGGCATGACACGGCGGACTTGTGTGGTCCCTGGTTTCTGTTTAGAATAGTGAGCGTTAACAGTGAGCGCTAACATTCAATCGACAACGAAGT-GCG-AGGCGCTTGA-------
BBPC_1564       ------------CGTTTACGATTGCGTGACACGTCGAACTTGTATAGTCGCCACTTTCTGTTTAGAATAGTGAGCGTTAACAGTGAGCGCTAACATTCCGTCGACAATGTGGTTGTG-AGGCGCTCGG-------
BBKW_1659       -------GCGCTGACGGATTGTTTCTTTGACACGCGAGTTTGTGCGGTTCGTAATTTACGTTTAGAATAGTGAGCGTTAACAGTGAGCGCTAACATTCAGTTGACAGAGTGGTTGCGTAGGCGCTTGG-------
BAD_1412        --------------TCATTGCTCCGATGACACGTCGGATTTGGTGCGTCACGGGTTTTTGCCTACAATAGTGAGCGTTAACAGTGAGCGCTAACATTCAGTTGACAGAGTGGTTGCG-AGGCGCTCGG-------
BSTER_1635      --------------TCATTGCTCCGATGACACGTCGGATTTGGTGCGTCACGGGTTTGTGCCTACAATAGTGAGCGTTAACAGTGAGCGCTAACATTCAGTTGACAGAGTGGTTGCG-AGGCGCTCGG-------
                                                       ***                    ** * *              ******* ****                 *

BSTEL_2135      ACGATCGGGCGGTGATGG---CCGGCCGGTCAGAAGGAGTCCA-C-------AG---AATGGCAGCAACGAACGCC---TCCGAGCAGGTCGCCACGATCGCGGAGAAGATTCGCGCCGGCAAGACCTCGCTGGG
BCAL_0892       ACGATCGGGCGGTGATGG--CCGGCCGGTCAGTGAGGAGTCA-C--------AACACAATGGCAGCAACGAACGATTCG---GAGCAGGTCGCCGCGATCGCCGAGAAGATCCGCGCCGGCAAGACCTCGCTGGG
BREU_1992       ---------------------CT-CCACGGCGTAAGGAA-A--------------GATATGGCAGCAATCGACAACGCGGCCGAACAGGTCGCTAACATCGCCGAGAAGATCCGCGCGGGCAAGACCACGCTGGG
BISA_1061       ----------------------CTCCACAGGGTAAGGAT-A--------------GATATGGCAGTTATCGACAACGCGGCCGAGCAGGTCGCTAACATCGCCGAGAAGATTCGCGCGGGCAAAACCACGCTGGG
BL0274          ----------------------GCTCCAAGGCAAAGGAA-A--------------GATATGGCAGCAACCGACAACGCGGCCGAGCAGGTCGCCCACATCGCCGAGAAGATTCGCGCTGGCAAGACCACGCTGGG
B8809_RS01645   ----------------------GCTCCAAGGCAAAGGAA-A--------------GATATGGCAGCAACCGACAACGCGGCCGAGCAGGCCGCCCACATCGCCGAGAAGATTCGCGCTGGCAAGACCACGCTGGG
BBIA_1211       ------------------ACTTCAGGCAAGAATAAGGAGTCCGC-----------ACTATGGCAGCAACCGTGAGCGACACCGAGCAGATCGCCGGCATCGCCGAAAAGATCCGTGCCGGCAAGACCGCGCTGGG
BBSC_2142       GATGACGGGCGGCGGCGGCCACCCGGCCACGTTAAGGAGCTCGA-A---------CGAATGGCAACAACGCACAATGACGCCGAACAGGTCGCAACGATCGCCGAGAAGATCCGCGCGGGCAAGACCTCGCTTGG
BDP_1924        ---------------------------A--CGCAAGGAGTTTGG-A---------ACTATGTCGCAA---------------GAGCCGGTTGCGTTGACCGCCGAGAAAATCCGCGCAGGCAAGACGAGCTTGGG
BMOU_0433       ---------------------------A--ACTAAGGAGATTGT-C---------ACTATGGCGCAAGAGCAGAAGCTTGACGACCGGGTCGCCTTGACTGCCGAAAAGATTCGCGCAGGCAAAACAAGCCTGGG
BBPC_1564       ---------------------------A--TGCAAGGAGTTTGA-A---------ACTATGGCGCAAGAGCAGGGACTTGACGATCGCATCGCGCTGACCGCCGAGAAAATCCGCGCTGGCAAGACGAGTCTGGG
BBKW_1659       ---------------------------A--CACAAGGAGTTTGA-C---------ACTATGGCGCAAGAGCAAGAACTTGACGACCAAGTCGCATTGACCGCCGAGAAGATCCGTGCCGGTAAGACGAGCCTAGG
BAD_1412        ---------------------------A--CGCAAGGAGTTCGTGA---------ACTATGACGCAA---------------GAGCAGGTCGCAGTGACCGCCGAGAAGATCCGCGCAGGCAAGACGAGCCTGGG
BSTER_1635      ---------------------------A--CGCAAGGAGTTCGTGA---------ACTATGACGCAA---------------GAGCAGGTCGCAGTGACCGCCGAGAAGATCCGCGCAGGCAAGACGAGCCTGGG
                                                  ****                    *** *                   ** *     **    *  ** ** ** ** ** ** ** ** **     * **
```

3. *araE - araB - araD - araA* (repression)

```
BIFANG_03633    GGCTGTAAATCAAGGTTTTGGGCGCCTGTTGACATTTATAAATGTGAGCGATAACATTTTTATTCAGCGAACGGGAAATATCACCGCAGACGAGTTTTCCGGATCGTCGTAGCGTACCGCGAACCTGCCTTCGGTG
BMERY_1744      GGCTA-AAAACAGGCTTTGTGAGATGCTTTGACATATCCGAATGTGAGCGATAACATTTCGAACTAGCGAACGGAAATCCTCATAGCAGACGGGAA-TCCGAGTCGCCGTAGCAAAGCCGCGAACCTGCCCCGGCC
                ****  *** ** * ***  *       ******* *   *******************  *   ********* **   ***  ******* *   ****  *** ******  * *    * *   *  ***

BIFANG_03633    ATAAGCCGCAGGCCACGTGTGAAGCGAAGCTGCTTCGGCAGGCTTACCTGCCGGTTACGGAACAGAAAAAGGAACAAAAATGACTGAGAACACTACGAGCGCTGTTAAAAAGCAGGGTTCAATGAAATATGTGGTC
BMERY_1744      CCAAGCCGTGGGTCCCGTGTGAAGCGAAGCTGCTTCGGCAGTCACGACTGCCGGTTACGGAACAGAAAAAGGAACAAT-ATGACTGAGAACGCTACGAGCGCTGTCAAGAAGCAGGGCTCAATGAAATACGTGGTC
                  ******  ** * ************************** *    ******************************  ************ ************* ** ******** *********** ******
```

4. *gap* (activation)

```
BSTEL_0869       ----------------CGGACGCCCTGACGCCCCTCATGTGAACGCTCACGGAATTTTCGGGATTTTCGGCTGAAACACGATGGAAGACGTTGCGTTTCGTACAGACGACGCACTAGAGTGGTC-GATGTTGGTA
BCAL_0662        ------------------GGTCCCGTGACGTCCGGAATGTGAACGCTCACAAAAAAACCTGTGTTTTCGCTGGAAACACCCGGCGAAACGTTGCCTGAAGTACAGGTGAGGCATTACAGTAAGGAATCGTTGGTA
BREU_1463        GTTCGGAAAAGCCCGCGAGAACAGGTGGCACAGCGGATGTGAGCGTTCACAAATTTTTGGTATTTTTTGCCCGAAACACACGTACGGCCATTGCTTTGTGTACACAGTCGGCATTACAGTAACA-ACTGTTGGTA
BISA_1483        ---GATGAACGTATGGCAACGCTCGCGCAAACAACAATGTGAACGCTCACAAAAAAATGGAATATTTTGCCCAAAACGCACGTTCAAACGTTGCTTTGTGTACACAGTCGGCATTACAGTAACA-ACTGTTGGTA
Bbr_1233         -ACGGGAAACCGCATATCGGTTGCCGGCGATCCACAATGTGAGCGCTCACAAAAAAACG-ACGTTTTTGCTCAAAACGCACGCTCAGACATTGCCATGTGTACAGAGTCGGCATTACAGTAGCA-ACTGTTGGTA
Blon_0900        GAGGAAAA-TCACATGCCGGTTGCGGGCAATCCGAATTGTGAGCGCTCACAAAAAATACGGCATTTTTGCCCAAAACGCACGCTGAAACGTTGCCATGTGTACAGAGTCGGCATTACAGTAGCA-ACTGTTGGTA
BL1363           GAGGAAAAATCACATGTCGGTTGCGGGCAATCCGAATTGTGAGCGCTCACAGAAAATACGGCATTTTTGCCCAAAACGCACGCTGAAACGTTGCCATGTGTACAGAGTCGGCATTACAGTAGCA-ACTGTTGGTA
B8809_RS05775    GAGGAAAAATCACATGTCGGTTGCGGGCAATCCGAATTGTGAGCGCTCACAGAAAATACGGCATTTTTGCCCAAAACGCACGCTGAAACGTTGCCATGTGTACAGAGTCGGCATTACAGTAGCA-ACTGTTGGTA
BBNG_00506       -----------------------TGCACAGACATATTTGTTAGCGTTAACGGAATATGGCCGTTTTATGCTCAAAGCAAGCGCGACACCGTTGCTC-TAGTACAGACGGCGCATTACAGTAGAC-ACTGTTGGTA
BBIA_2113        GGCCGTCAAGCGGCATCCACATCCTGCGCAAGTCTTATGTGAGCGTTCACGGAAAGTTTGCCCGATACGCGAAAACACGCCCGCATATCGTTGCGAAAGGTACAGGCGACGCATTAGAGTGGGA-CATGTTGGTA
BBSC_1757        -------------------------GGGGCAGTGGAGTGTGAGCGCTCACAAACCCAACATCCATTCGTGGAAACACGCCCGC-AAAACGTTGCCGGAAGTACAGGCGAGGCATTAGAGTGGGC-TGTGTTGGTA
BDP_1514         -----------------------------AAACTCATTGTGAGCGTTCACGGAAACGACGTCGTTTTCTGAAACACACGACCGCCATACGTTGCCCTAAGTACAGGTGACGCACTAAAGTGGGC-GGTGTTGGTA
BMOU_0196        -----------------------------AAACCTACCGTTAGCGTTCACGCAAATGGCGTCGTTTTCTGAAACACACTCCCGCCATGCGTTGCCCTAAGTACAGGTGACGCACTAAAGTGGGC-GGTGTTGGTA
BBPC_1247        -----------------------------ACATGCAGCGTGAGCGCTCACAGAAAATACGACGTTTTC-TGAAACACACGCGCGAATGCGTTGCCTTAAGTACAGGTGACGCACTAAAGTGGGT-GACGTTGGTA
BBKW_1382        -----------------------------ACATGCAGCGTGAGCGCTCACAGAAAATACGACGTTTTC-TGAAACACACGTGCGAGTGCGTTGCCTTAAGTACAGGTGACGCACTAAAGTGGAT-GACGTTGGTA
BAD_1079         -----------------------ATGCGGGACATGCATGTGAGCGTTCACTGAAAATCGCACTGCATTTTGGAACACGCGCCTCGGAACGTTGCCCTAAGTACAGGTGACGCACTACAGTGGAC-GGTGTTGGTA
BSTER_1443       -----------------------ATGCGGGACATGCATGTGAGCGTTCACTGAAAATCGCACTGCATTTTGGAACACGCGCCTCGGAACGTTGCCCTAAGTACAGGTGACGCACTACAGTGGAC-GGTGTTGGTA
BBOMB_0776       -------------------CGTCAGACGTCCGATTCTTGTTAGCGGTAACATGGTTTGGTCGGTTTTGATGAAAAAACATGGGAATCGGCTTTCCGCGGTACAGCGCGCACAGTAAAATAGGA-GCCGTTGGTA
                                                      ** * ** * **                                      *       *****       ** ** * *         *******

BSTEL_0869       AACAATGGCTCCGGCGTGCCCA-----------------------------------GCGTGC-CGGC--GTCACCC--TACAAGGGAGAATTACATGACAGTTAAGATTGGTATCAACGGCTTCGGTCGCATCG
BCAL_0662        AACAATGGTTCCGTGTGCGT-CC--------------------ACGT--------------GC-CCGG--GGCCTTC--CCCAAGGGAGAATTACATGACAGTTAAGATTGGTATCAACGGCTTCGGTCGCATTG
BREU_1463        AACAACGGCGCGGTATGCGCACA--------------------GCGC--------------AT-GTCC--GCCACCC--TACAAGGGAGAATTACATGACAGTTAAGATTGGTATCAACGGCTTCGGTCGCATTG
BISA_1483        AACAATGGCGTGGTGTGCCACA---------------------GCAC--------------AT-TACC--GCCACCC--TACAAGGGAGAATTACATGACAGTTAAGATTGGTATCAACGGCTTCGGTCGCATTG
Bbr_1233         AACAATGGCCCGGTGTGCCA-GA--------------------GCGC--------------AC-CACG--GGCACCC--TACAAGGGAGAATTACATGACAGTTAAGATTGGTATCAACGGCTTCGGTCGCATTG
Blon_0900        AACAATGGCCCGGTGTGCCA-AA--------------------GCGC--------------GC-CAAG--GCCACCC--TACAAGGGAGAATTACATGACAGTTAAGATTGGTATCAACGGCTTCGGTCGCATTG
BL1363           AACAATGGCCCGGTGTGCCA-AA--------------------GCGC--------------GC-CAAG--GCCACCC--TACAAGGGAGAATTACATGACAGTTAAGATTGGTATCAACGGCTTCGGTCGCATTG
B8809_RS05775    AACAATGGCCCGGTGTGCCA-AA--------------------GCGC--------------GC-CAAG--GCCACCC--TACAAGGGAGAATTACATGACAGTTAAGATTGGTATCAACGGCTTCGGTCGCATTG
BBNG_00506       AACAAAGGCCGTAGCGCATCCAT--------------------GCGC--------------AA-ACGG--CCTACCC--TACAAAGGGAGAACACATGACAGTTAAGATTGGTATCAACGGCTTTGGTCGTATCG
BBIA_2113        AACAATGGCCCCAGCGTGCCTA----------------------------------GGCATGTGCCGG--GTCACCC--TACAAGGGAGAATTACATGACAGTTAAGATTGGTATCAACGGTTTCGGTCGTATCG
BBSC_1757        AACAATGGCCCCATGTGTTCCA-----------------------------------ATGCGCGCGGG--GTCACCC--TACAAGGGAGAATTACATGACAGTTAAGATTGGTATCAACGGCTTCGGTCGCATCG
BDP_1514         AACAATGGCTTCATTGCGCCTCG--------------------CGCA--------------TG-TGTG--GCCTACC--CTAAAGGGAGAATTACATGACAGTTAAGATTGGTATCAACGGCTTTGGTCGTATCG
BMOU_0196        AACAATGGCTTCATTGCGCCCCG--------------------CGCA--------------TG-TGTG--GCCTACC--CCAAAGGGAGAATTACATGACAGTTAAGATTGGTATCAACGGCTTTGGTCGTATCG
BBPC_1247        AACAATGGCTTCTGTGTGCCCAG--------------------CACA--------------CG-TGTG--GCCTACC--CTAAAGGGAGAATTACATGACAGTTAAGATTGGTATCAACGGCTTTGGTCGTATCG
BBKW_1382        AACAATGGCTTCTGTGTGCCCAG--------------------CACA--------------CG-TGTG--GCCTACC--CTAAAGGGAGAATTACATGACAGTTAAGATTGGTATTAACGGCTTTGGTCGTATCG
BAD_1079         AACAATGGCTTCAGTGTGCCTGG--------------------CACA--------------CG-CGTG--GCCTACC--CTAAAGGGAGAATTACATGACAGTTAAGATTGGTATTAACGGCTTTGGTCGTATCG
BSTER_1443       AACAATGGCTTCAGTGTGCCTGG--------------------CACA--------------CG-CGTG--GCCTACC--CTAAAGGGAGAATTACATGACAGTTAAGATTGGTATTAACGGCTTTGGTCGTATCG
BBOMB_0776       AAAATAGCCGTCGAGCTCCAACTTACAA-----------CAAGATGC--------------GC-CACG--GCTAAAT--TCAAAGGGAGAATTACATGACAGTAAAGATTGGTATCAACGGCTTCGGTCGAATCG
                 ** *  *                                                                     ** **    *  ********** *********** ***** ** ***** ** *
```

5. *eno* (activation)

```
BMIN_1358       ---------ATGGCATTGTGAGCGTTCACGAAC--CGCCGCGAC-GGCGA-ACGGTCTAGTCCATAAAGCCCGGTATCCTTAGAA-CCGTTATCGCA--A-CCTACG--T-TGTAAGGAGCAGTTGTGGCAGCTA
H0424_RS03195   -GTTGAATCAAAATATTGTGAACGTTCACGGCATAAATACGCTGGTACTC-AATATAACGTCCATAAAACGCACTAATCTTAAAA-GAGTCATCACA--A-CCCATAGGT-ATAAAGGAGTAGTTGTGGCAGCTA
BBOU_0409       -TATCTCGCACGCGGCTGTGAACGCTCACAGGGAACAGTGAGTGAAATCG-ACGATATCGTCCAACAATTCCACTATCCTTGACA-TTGTCATCACA--A-CCTGA---G-CAAAAGGAGTAGTTGTGGCAGCAA
D805_0822       -TGAAGCGCACGCGGCTGTGAACGCTCACAGGAAACAGTGAGTGAAATCG-GCGATATCGTCCAACAATTCCACTATCCTTGACA-GTGTCATCACA--A-CCTGA---G-CAAAAGGAGTAGTTGTGGCAGCAA
BITS_1198       --ATGGCGTCGAAAACTGTGAACGCTCACACACGCCATGCAGCACAATCA-CAGTTATCGTCCACAAAGCCCACTATTCTTGGAA-ACGTCAACACA--A-CCTAA--TT-GCTAAGGAGTAGTTGTGGCAGCTA
BSTEL_0380      --GGTGAACAGTGGGATGTGAACGCTCACAACACGCAATGCGGA-ACCCT-TGCGCGTAGTCCATCAATCACACTATTCTTGACG-ATGTCATCACA---ACCTAACG---TTAAAGGAGTAGTTGTGGCAGCAA
BCAL_0819       --GTATTTCCAATAACTGTGAGCGCTCACAACACGCTTTGCGCC-CCTCT-TGCGCGTAGTCCACAAATCGCACTATTCTTGACG-ATGTCATCACA---ACATAACG---TTAAAGGAGCAGTTGTGGCAGCAA
BREU_1061       -GCGTTCCGTCAGGAATGTGAGCGCTCACACCAT-TTTTGGGCG-TGTCG-TTCACAACGTCCACAAATCGCACTAGTCTTAGGA-CTGTCATTAGA--TACACAACCGT-TTTAAGGAGTAGTTGTGGCAGCAA
BISA_1296       CGGAGAACAAA-TAAATGTGAGCGCTCACACTAT-TTTTGGGCG-TGTCG-TTCACAACGTCCACAAATCGCACTAGTCTTATGA-CTGTCATTAGA--TACACAACCGT-TTTAAGGAGTAGTTGTGGCAGCAA
Bbr_0725        CGCGGCACACCGACATTGTGAGCGCTCACATCAAATTTTGGGCG-TGTTG-TTCACAACGTCCACAAATCGCACTACTCTTATGA-ATGTCATTCAAGATACACAACCGT-TTTAAGGAGTAGTTGTGGCAGCAA
Blon_1836       GGCGGCACACTGGCATTGTGAGCGCTAACACTAAATTTTGGGCG-TGTTG-TTCACAACGTCCACAAATCGCACTACTCTTAAGA-ATGTCATTCAAGATACACAACCGT-TTTAAGGAGTAGTTGTGGCAGTAA
BL1022          GGCGGCACACTGGCATTGTGAGCGCTAACGCTAAATTTTGGGCG-TGTTG-TTCACAACGTCCACAAATCGCACTACTCTTAAGA-ATGTCATTCAAGATACACAACCGT-TTTAAGGAGTAGTTGTGGCAGTAA
B8809_RS02940   GGCGGCACACTGGCATTGTGAGCGCTAACGCTAAATTTTGGGCG-TGTTG-TTCACAACGTCCACAAATCGCACTACTCTTAAGA-ATGTCATTCAAGATACACAACCGT-TTTAAGGAGTAGTTGTGGCAGTAA
BBNG_00642      --TATATCCAGCCGAATGTTAGCGCTCATATTATTTTTCCGAGA-AATTC-TGGCACTCGTCCACAAAAGGCATTATTCTTAAGT-CTGTCATCACA--A-CCTAACAGT-CTTAAGGAGTAGTTGTGGCAGCTA
BBIA_2197       -GTGTCGTCACAACGATGTGAGCGCTCACATTTTCGTGTCGCAT-CCCCT-TGCGTCTCGTCCATCAAACCCGGTATTCTTAAGG-CTGTCATCACA---ACCTATACG--TTAAAGGAGTAGTTGTGGCAGCTA
BBSC_1435       --TGCGCGCACCTGAATGTGAGCGCTAACAAACTTGTTGCCGAA-AAGGG-TGTCGGTCGTCCATAAATCCCACTATTCTTAGGA-CTGTCATCACA---ACCTAACA---TTAAAGGAGTAGTTGTGGCAGCAA
THER5_1063      -TATCTCGCGCGTGGCTGTGAACGCTCACAGGAAACAGTGAGTGAAATCG-GCGATATCGTCCAACAATTCCACTATCCTTGACA-GTGTCATCACA--A-CCTGA---G-CAAAAGGAGTAGTTGTGGCAGCAA
BDP_0870        -ATGTCCGCAGATGTATGTGAGCGTTCACATGATTCGTGTCGGAAAAGTT-TGCGCTACGTCCACAAAACGCACTATTCTAGGCA-TTGTCATCACA---ACCTAACGTT-TATAAGGAGTAGTTGTGGCAGTAA
BBPC_0725       -ATCCGACACGATTTGTGTGAGCGTTCACACAAATCGTGTCGGATAAGTT-TGCCATACGTCCACAAAACGCACTATTCTAGGCA-ATGTCATCACA---ACCTAACGTT-TATAAGGAGTAGTTGTGGCAGTAA
BBKW_0740       -GTCCGACACGATTTATGTGAGCGTTCACATAAATCGTGTCGGATAAGTT-TGCCATACGTCCACAAAACGCACTATTCTAGGCA-ATGTCATCACA---ACCTAACGTT-TATAAGGAGTAGTTGTGGCAGTAA
BAD_0645        ---ATGCAAAAAAGAGTGTGAGCGTTCACATGGAACATGCGGGCAAAGTT-TGGCATACGTCCATAAATCGCACTATTCTTGGCA-TTGTCATCACA---ACCTAACGTT-TATAAGGAGTAGTTGTGGCAGTAA
BSTER_1777      ---ATGCAAAAAAGAGTGTGAGCGTTCACATGGAACATGCGGGCAAAGTT-TGGCATACGTCCATAAATCGCACTATTCTTGGCA-TTGTCATCACA---ACCTAACGTT-TATAAGGAGTAGTTGTGGCAGTAA
BCHO_1674       ACGGGGCGCGCCGGGGTGGGAGCGCTCACAGTTTTCGCCGACGG-GAAGTT-GCGAATCGTCCAACAACGGCATTACTCTTGCAG-ATGTCATCACA--A-CCCGTACA--CACAAGGAGCAGTTGTGGCAGCAA
AH67_05625      GGGCCGCCCGATAGATTGTGAGCGCTCACAAAGAATGGTGGGT--CCGGC-GCGCACTCGTCCAATAAGCCCACTACTGTTGGAGACTGTCAACACA---AC-CTATGCA-CATAAGGAGTAGTTGTGGCAGCAA
BMAGN_0589      TAGGAAGAACGGAGGCTGTTAACGCTCACATTTTTCTGATAGC--AAGGT-TGCCACTCGTCCACAAAACCCACTACGCTGTGAA-GTGTCATCACA---AC-CT--ATACATAAAGGAGTAGTTGTGGCAGTTA
BSAE_0901       --AGGGGATTCGCCGATGTGAGCGCTAACGAAAGTTCTTCCGCG-TGTCTTCGCCCAACGTCCATCAAACCCACTATCCTTAACA-GTGTCATCACA--A-CCCAACGT--CGTAAGGAGTAGTTGTGGCAGTAA
BPSY_1734       GGATAGGCATCCGACTTGTTAGCGCTAACATAAAAATTGGCCCC-ACTCAG-CGCCATCGTCCAACAAACGCACTACTCTTATGA-GTGTCAGCACA--A-CATACATGT-CTTAAGGAGAAGTTGTGGCAGCAA
BISU_0542       ------CATCGTTGGATGTGAACGCTCCCAGCGGGCGGTGCCGTCAGAGC-GCGCTGTAGTCCAACAAACACACTATTCTTGACG-GTGTCATACAA---CCA---ACGC-TTGAAGGAGTAGTTGTGGCAGCAA
BBOH_1126       GAGCTTGAGCATAGTATGTGAGCGCTAACATAAAACCAACAT-CTTTTGT-TGGCTTCCGTCCTTGAGAACTACTATTCTTGAAC-ATGGTAATAAC---ACA---AAGC-TGTAAGGAGCGATTGTGGCAGCAA
BBOMB_0226      GGTTTCCCACGTTCTATGTTAACGCTAATATAAATTATGAT-T--TCCTT-GCACGCTCGTCCAACTTTGCAACTAAACTGGTAG-CTGTTAGTACA---AA-CA--AGC-TCGAAGGAGTAATTGTGTCTGCAA
                                **  * ** *                                 ****           **   *        *  *                      ******   ***** * *  *
```

6. *pyk* (activation)

```
BITS_1163    TGTGGCACGATTCATTTATAAAACGTGGAAAACCGTCATTGTTAGCGCTCACAACACGCCGAGTGCATAGGA-TGACTTGGA-GAAGTGAAGCAGGTAGACTTGTTCT-TGGAAGTGGGT-----CGTTCGCGGCTCA
BSTEL_0427   ---------------------GTATTGCGGCGATTGGGTTGTGAGCGTTAACAACACACCGAGAGGAAAAC-TGGAAGGAAC-GGATGAAAGCTAGTACACTGTTCCGTGGAAAACGTAG-----GTCGTCCGGCCTG
BCAL_0786    ---------------------TTGTTGCGGCCATTGGGTTGTGAGCGCTAACAACACGCCTTGTGAAAAAC-TGGATTCACC-GGAGGGAACCTAGTACACTGCTGTAGTGGAAAACGGA-----GCCATTCCG-GTT
BREU_1094    ---------------------CC-CAATGGCCACAACAATGTGAGCGCTCACAACACACCGGCGAAAAAAT-TTGTCTTGCGTCGGTGAGCGCTAGTAGACTGAAACA-CGGCAAGTGAG-----GCTTTCCGCCTCG
BISA_1328    ---------------------CC-CAATGGGCAGAACAATGTGAGCGCTCACAACACACCGAGTAAAAAAC-TTGTCATGCGTCGGTGAGCGCTAGTAGACTATTGCA-CGGCAAGTGAG-----GCTTCAAGCCTCG
Bbr_0757     ---------------------CC-CAATCGGCGCAACAGTGTGAGCGCTCACAACACACCGGGGAATAAAC-TTGTCATGAGCCGGTCTGCGCTAGTAGACTGGGCTG-CGGCAAGTGAG-----GCAATCGCGCCTC
Blon_1745    ---------------------CC-CAGTCAACACAACAATGTGAGCGCTCACAACACACCGTGAAGAAAAC-TTGTCATGAGTCGGCTCGCGCTAGTAGACTGAGTTT-CGGCAAGTGAG-----GCGATCGCGCCTC
BBSC_1400    ---------------------CC-TTTTCCCGACATAATTGTTAGCGCTCACAACACACCCCGTTCCGTAC-TGGAGAAGT-TGGGGCCAAGCTAGTAGACTATGCCG-TGGAAGCCGGG-----CCGTCAGGTCCGG
THER5_1025   ---------------------AACGCGACATGAAGCTGATGTGAGCGTTCACAACACAGTTGCCTGAATGG-TTGGCGCATGGTGGGGGAACCGAGTAGACTAGCACA-CGGAAACAGGA-----TTCTCACCTGTTC
BDP_0904     ---------------------TCGTTCTCCCGACAAAAATGTGAGCGCTCACAACACGCCTCTGTCCGGAG-TGGAAGTGAG-CGCTTTTTGCTAGTAAACTATGCCATTGGAAACAGGG-----TCGTCAGATCCAA
BMOU_0847    ---------------------TCATTCTCCCGAAAGAAATGTGAGCGCTCACAACACACCTCGGTCCGGGG-TGGAAGTGAG-CGCTATTTGCTAGTAGACTATGCCATTGGAAACAGGG-----TCGTCAGATCCAA
BBPC_0760    ---------------------TCTTTT-TATCAAAAAAATGTGAGCGCTCACAACACGCCTCGTTTCAGGG-TGGAAGTGAG-CGCTTTTTGCTAGTAGACTGGTCGGTTGGAAACAGGG-----TCGTCAGATCCAA
BBKW_0773    ---------------------TCTTTT-TAGCAAAAAAATGTGAGCGCTCACAACACGCCTCGTTTCAGGG-TGGAAGTGAG-CGCTTTTTGCTAGTAGACTAGTCGGTTGGAAACAGGG-----TCGTCAGATCCCA
BSTER_1676   ---------------------TCTATTCTCCTACGAAATTGTGAGCGCTCACAACACACCTAGTTCTGAGG-TGGAAGTGGG-CGCTTTTTGCTAGTAAGCTATGCCATTGGAAACAGGG-----TCGTCAGATCCAA
BCUN_0324    ---------------------CATAGCGGGATCAATGTTTGTTAGCGCTCACAAAAAAACGCCGGAGCGGC-TTGCGTGATG-GAGGTATTGCGG-TAAGCTGGGTGATGGCATTCGCGG-----GGTTGCCCG-CGG
BMAGN_0625   ---------------------CTTATATTTTCCACCGATTGTTAGCGCTAACAGAAAAACGCTTGCGCTTCG-GGCGTGG-CGCGGTTTTCGCGACTAAACTAGTAGC-CGATCACCGGG-----AGTTACCACCCGA
BSAE_0869    ---------------------CGATTGCGCCCACCGGAATGTGAGCGCTAACAAACACACCGTGCGGAAAAGTGGCTCCGTG-CAGGTTTTCCTAGTAAACTGTCGTG-TGGCAACTGGG-----TCGTCAGACCCCC
BPSY_1700    ---------------------CATTGGGCTCACATCAATTGTGAGCGCTAACAACACACCGGGCCGATTCGCTGTCTTCCGT-CGCCGATTGCTAGTAGACTCGGTTT-TGGAAGACGAG-----GGGATATCTACCC
                                                    *** **** * ***                                       *    **   **

BITS_1163    ATCAA--C---AA-CAATCCAAGATAGGCAGGCATGTATGCGCAAAGCCAAAATCGTCGATACCATCGGTCCCGCTACCGAATCTTATGACAACCTCCTCAAGCTGGTCGAGGCAGGTAT
BSTEL_0427   CG---TCC---CT-TGCAATAGTTAGGAATAGGCAACATGCGCAAAGCCAAGATCGTCGACACCATCGGTCCCGCCACCGAATCCCTCGAAGGCATCACCAGCCTCGTCGAGGCCGGCAT
BCAL_0786    CC---GTC---CG-TTGCAAAGTTAGGAATAGGCAACATGCGCAAAGCCAAGATCGTCGACACCATCGGCCCCGCCAGCGAGTCCCTTGAAGGCATCACCAGCCTCGTTGAGGCCGGCAT
BREU_1094    AACCA--C---A---AGAAAAGGATAGGCAGGCATTCATGCGTAAAGCCAAGATCGTAGACACCATCGGTCCTTCCACCGAGGATTACGACAACCTCCTCAAGCTCGTCGAAGCCGGTAT
BISA_1328    AACCA--A---CA--AAGAAAAGATAGGCAGGCATTCATGCGTAAAGCCAAGATCGTAGACACCATCGGTCCCGCTACCGAGGATTACGACAACCTCCTCAAGCTCGTTGAAGCTGGTAT
Bbr_0757     GAACCA-A---AAAAGAACAAGAATAGGCAGGCATTCATGCGTAAAGCCAAGATCGTAGACACCATCGGTCCTTCCACCGAGGATTACGACAACCTTCTCAAGCTCGTCGAAGCCGGTAT
Blon_1745    GAACCA-A---AT-TGAACAAGAATAGGCAGGCATTCATGCGTAAAGCCAAGATCGTAGACACCATCGGTCCTTCCACCGAGGATTACGACAACCTCCTGAAGCTCGTTGAAGCCGGTAT
BBSC_1400    GTGTA--C---A---AGATAAGAATAGGCAGGCATTCATGCGTAAAGCCAAGATCGTAGATACCATCGGTCCCGCTAGCGAGGATTACGACGAGCTCCTGAAGCTCGTCGAAGCCGGTAT
THER5_1025   GATAGA-AAAGTA-AGTTGAAACAGAGGCAGGTATTCATGCGTAAAGCAAAGATCGTCGACACGATCGGTCCAGCCACCGAGTCCCTCGAAGGCATCACCAAGCTCGTCGAAGCCGGTAT
BDP_0904     ATCAA--A---TT-AGAACAGGAATAGGCAGGCATACATGCGTAAAGCCAAGATCGTAGATACCATCGGTCCCGCTACCGAGTCTCTGGAAGGCATCACCAGCCTCGTCGAAGCAGGTAT
BMOU_0847    ATCAA--G---TT-AAAACAGGAATAGGCAGGCATACATGCGTAAAGCCAAGATCGTAGATACCATCGGTCCCGCTACCGAGTCTCTGGAAGGCATCACCAGCCTCGTCGAAGCCGGTAT
BBPC_0760    AATCA--A---TA-TCAACAAGAATAGGCAGGCATTCATGCGTAAAGCCAAGATCGTAGATACCATCGGTCCAGCTACCGAGTCCCTGGAAGGCATCACCAGCCTTGTCGAAGCAGGTAT
BBKW_0773    AATCA--A---TA-TCAACAAGAATAGGCAGGCATTCATGCGTAAAGCCAAGATCGTAGATACCATCGGTCCAGCTACCGAGTCCCTGGAAGGCATCACCAGCCTTGTCGAAGCAGGTAT
BSTER_1676   AATCA--A---TA-TCAACAAGAATAGGCAGGCATTCATGCGTAAAGCCAAGATCGTAGATACCATCGGTCCCGCCACCGAGTCCCTGGAAGGCATCACCAGCCTTGTCGAAGCAGGTAT
BCUN_0324    AG---TGA---CA-ACAGAAAGAACAGGTAGGCATACATGCGTAAAGCCAAGATCGTAGACACCATCGGTCCCGCCACCGAATCGCTCGAGAATCTCACGAAGCTCGTCGAGGCGGGCAT
BMAGN_0625   TGTTAC------C-TATGTAGAAAGTGGATAGCATTCATGCGTAAAGCAAAGATTGTGGACACCATTGGTCCAGCTACCGAATCTCTCGAAAACCTCACCAAGCTTGTGGAAGCCGGTAT
BSAE_0869    ACACG---------TCAAGAAGGATAGGCAGCCAT--ATGCGTAAAGCCAAGATCGTAGATACCATCGGTCCCGCCACCGAATCGCTCGAGAACCTCACCAAGCTCGTCGAAGCGGGTAT
BPSY_1700    CCCAACCA---TC-AAATAGGAAATAGGCAGGCATTCATGCGCAAAGCCAAGATTGTAGACACCATCGGACCAGCCACAGAATCGTTGGAGAACCTCACCAAGCTCGTCGAAGCGGGTAT
                                          *          ***** ***** ** ** ** ** ** ** ** **  * *  **        **      *     *    * ** ** ** ** **
```

7. *tkt - tal* (activation)

```
BSTEL_0929   ---------------GACACATATTGTGAACGCTAACAGAACAGAAGTTACCAAA-ACACGCCAAAATCAAAACGCTACGTCGCGAAATG-AAGGTAGCATGGGTAACGCAATGTGAGGGCGACA-GCGCCCGGCACA
BCAL_0272    ---------------GGCACATTCTGTGAACGCTAACGGAACAGAAGCTACCAAA-ACACGCCAAAATCACACCACTACGTCGTGAAATG-ACGGTAACATAGGCATCGCAATGTGAGGGCGTCTTGCGCTCGGCACA
BREU_0025    ---------------GATGTGAATTGTGAACGCTAACAGCACACAAACTACCAAA-ACACGCCAAATATGCACCACAAACGTCGTCTTGCGACGCTAGCATGGAAAACGCAATGTGAGGGCGTCATGCGCCCGGCACA
BISA_0603    ---------------CATGTTAATTGTGAACGCTAACAGAACACAACTTACCAAA-ACACGCCAAATATGCACCACAAACGTCTTCTTGCGACGTTAACATGGGAAACGCAATGTGAGGGCGTCGTGCGCCCGGCACA
Bbr_1003     ---------------GATGTGAATTGTGAACGCTAACAGAACACAAACTACCAAA-ACACGCCAAAATTGCACCATAACGTCGCAAACCA-TCGCTAGCATGGTAAGCGCAATGTGAGGGCGTCAAGCGCCCGGCACA
Blon_1096    ---------------AATGTTCACTGTGAACGTTAACAGAACACAAACTACCAGA-ACACGCCAAAATTGCACCACAACGTCGCAAACCG-ACGGTAGCATGGTAATCGCAATGTGAGGGCGTCAAGCGCCCGGCACA
BL0716       ---------------AATGTTCACTGTGAACGCTAACAGAACACAAACTACCAAA-ACACGCCAAAATTGCACCACAACGTCGCAAACCG-ACGCTAGCATGGTAATCGCAATGTGAGGGCGTCAAGCGCCCGGCACA
BBNG_00949   CGTCGGCATGAGTCAGTCAATACTTGTGAGCGCTAACATAACATAATCTACCAAA-ACACGCCAAAAGCGAACCACTAAGTCTGTTCACA-ACGTTAGTATGGGATT-GCAATGTGAGGGCATGCATGGCGCGGCATA
BBIA_1485    ACACAACGTACCATCCACGTTCATTGTGAACGCTAACAGCACACAAACTCACAAA-ACACGCCAAAAACCACACCGCTTAGTCGGTTCGCACGGTTATTATG-TAAGGGCAATGTGAGGGCATGCATGGCATGGCATA
BBSC_1462    GCGTAATGCCACATTTTTGTTCACTGTGAACGCTAACAGCACACAAGTTAACAAAAACACGCCAAAATCGCACCGCTAAGTCGGTTGAGG-CGGTTAGTATG-TAAGAGCAATGTGAGGGTATGCATGGCCCGGCACA
BDP_1145     GTGCAAATGCACGGAAATGTTCATTGTGAACGCTAACAGAACACAACTTACCAAA-ACACGCCAAAATATCACTGCTACGTCATCGCGCA-TCGCTACTATGGGATACGCAATGTGAGGGCGTCAAGCGCTCGGCACA
BMOU_1044    GCGCAAACGCACGGGAATGTTCATTGTGAGCGTTAACAGAACACAACTTACCAAA-ACACGCCAAAATTTCACTGCTACGTCACCTCACA-TCGCTACTATGGAATACGCAATGTGAGGGCGTCAAGCGCTCGGCACA
BBPC_0945    GAGCAATCGCACGAGAATGTTAACTGTGAGCGTTAACAGAACACAAACTACCAAA-ACACGCCAAAAATCCACCACAACGTCACCTCACA-TCGCTACTATGGAAAACGCAATGTGAGGGCGTCGAGCGCTCGGCACA
BBKW_1047    GGACGATCGCACGAGAATGTTAATTGTGAGCGTTAACAGAACACAAACTACCAAA-ACACGCCAAAAATCCATCACACGTCACCTCACG--TCGCTACTATGGAAAACGCAATGTGAGGGCGTCGAGCGCTCGGCACA
BAD_0829     TCTGAAGTTCACGCGAATGTTAATTGTGAGCGCTAACAGCACACAAACTACCAAA-ACACGCCAAAATTTCACCACAACGTCACCTCACA-TCGCTACTATGGGAAACGCAATGTGAGGGCGTCGAGCGCTCGGCACA
BSTER_1013   TCTGAAGTTCACGCGAATGTTAATTGTGAGCGCTAACAGCACACAAACTACCAAA-ACACGCCAAAATTTCACCACAACGTCACCTCACA-TCGCTACTATGGGAAACGCAATGTGAGGGCGTCGAGCGCTCGGCACA
BSAE_1261    --------------GCGGCTGAATTGTGAGCGTTAACATCGCGAAATGGCCGAAA-ACACGCGAACAATGCCTTGTCAGGTCGGTCTTGA-AAGTTAGGATG-TAAGAGCAATGTGAGGACGTTCATGGCCCGGCACA
BISU_1589    ---------AAA-TATGCGGATTCTGTGAACGCTCACGAACCGTAAACCGGCCGA-ACACGCCAACGCCGCGGCGTCAAGTCGTCTGCGA-AAGCTACCATGA-AGGGGCAATGTGAGGGCATATATGGCGCGGCATA
                                     ***** ** * **    *  **        * ****** **                            * **  **       ***********        **  **** *

BSTEL_0929   AGCC--GGATA-CG--CG--G--CCCGACC-AAGCGGAAGGAAA---GACA----TC-CATGACCGAATTCAAGGAGACCGAGCTGGACGAGCGCGCCATCAAGATGGCCAAGGTC
BCAL_0272    AGCC--GGATA-CG--CG--G--CCCGACC-AATTGGAAGGAAA---GTAA----TC-CATGACCGAATTCAAGGAGACCGAACTGGACGAACGCGCCATCAAGATGGCGAAGGTC
BREU_0025    AGCC--GGATA-CG--CG--G--CCCGACC-AATTGGAAGGAAA---GTAA---ATC-CATGACCGAATTCAAGGAGACCGAGCTCGACGAGCGTGCCATCAAGATGGCAAAGGTC
BISA_0603    AGCC--GGATA-CG--CG--G--CCCGACC-AATTGGAAGGAAA---GTAA---ATC-CATGACCGAATTCAAGGAGACCGAGCTCGACGAGCGCGCCATCAAGATGGCAAAGGTC
Bbr_1003     AGCC--GGATA-CG--CG--G--CCCGACC-AATTGGAAGGAAA---GTAA----TC-CATGACCGAATTCAAGGAGACCGAGCTCGACGAGCGCGCCATCAAGATGGCGAAGGCC
Blon_1096    AGCC--GGATA-CG--CG--G--CCCGACC-AATTGGAAGGAAA---GTAA----TC-CATGACCGAATTCAAGGAGACCGAGCTCGACGAGCGCGCCATCAAGATGGCGAAGGTC
BL0716       AGCC--GGATA-CG--CG--G--CCCGACC-AATTGGAAGGAAA---GTAA----TC-CATGACCGAATTCAAGGAGACCGAGCTCGACGAGCGCGCCATCAAGATGGCGAAGGTC
BBNG_00949   AGCC--GAAATGCGTGGT--G--CCCGAAG-TATTGGAAGGAAA---GTA-C--ATC-CATGACCGAATTCAAGGAGACCGAGCTGGACGAGCGCGCCATCAACATGGCCAAGGTC
BBIA_1485    AGCC--ATGCG-CG--TG--GTGTCCGAAG-TATTGGAAGGAAA---ACTAC--AGA-CATGACCGAATTCAAGGAAACCGAACTGGACGAGCGCGCCATCAAGATGGCCAAGGTC
BBSC_1462    AGCC--GGACG-CG--TG--GTACCCGAAG-TATTGGAAGGAAA---ATTAC--AGA-CATGACCGAATTCAAGGAAACCGAACTGGACGAACGCGCCATCAAGATGGCCAAGGTC
BDP_1145     AGCC--GGATA-CG--CG--G--CCCGACC-AATTGGAAGGAAA---GTAA----TCCTATGACCGAATTCAAGGAGACCGAGCTGGACGAGCGCGCCATCAAGATGGCCAAGGTC
BMOU_1044    AGCC--GGATA-CG--CG--G--CCCGACC-AATTGGAAGGAAA---GTAA----TCCTATGACCGAATTCAAGGAGACCGAGCTGGACGAGCGCGCCATCAAGATGGCCAAGGTC
BBPC_0945    AGCC--GGATA-CG--CG--G--CCCGACC-AATTGGAAGGAAA---GTAA----TC-CATGACCGAATTCAAGGAGACCGAGCTGGACGAGCGCGCCATCAAGATGGCCAAGGTC
BBKW_1047    AGCC--GGATA-CG--CG--G--CCCGACC-AATTGGAAGGAAA---GTAA----TC-CATGACCGAATTCAAGGAGACCGAGCTGGACGAGCGCGCCATCAAGATGGCCAAGGTC
BAD_0829     AGCC--GGATA-CG--CG--G--CCCGACC-AATTGGAAGGAAA---GTAA----TC-CATGACCGAATTCAAGGAGACCGAGCTGGACGAACGCGCCATCAAGATGGCCAAGGTC
BSTER_1013   AGCC--GGATA-CG--CG--G--CCCGACC-AATTGGAAGGAAA---GTAA----TC-CATGACCGAATTCAAGGAGACCGAGCTGGACGAACGCGCCATCAAGATGGCCAAGGTC
BSAE_1261    AGCC--GAAGG-CG--TG--GCGTTCGACG-TATTGGAAGGAAA---GTAGA--ATC-TATGACCGATAACACGTGGTCTGAATTGGACGAGCGCGCCATCAAGATGGCGAAGGTG
BISU_1589    AGCC--GTATG-CG--TGATG--CTCGCAG-TATTGGAAGGAAA---GTAGATCACC-TATGACCGATTTCACTTGGTCTGACTTGGACGAGCGCGCTGTGAAGATGGCGAAGGCC
             ****        **      *    **     *  *********               ********   **      * **  * ***** ** **  * ** ***** ** *
```

8. *ldh* (activation)

```
                         Crp site             AraQ site        Crp site (not conserved in all strains)
BBOU_0652                        CGTTTGCCCCGCACACAGTGTGAACGCTCTCAAATTCATC------------TCGTACCGGTTCGCCCG--CAAAGCGTTT-T--CCCCGGAGAAACGTCACGGAGAACCACTAGAGTAGAAGTGTT
D805_0597                        TACTTAATACGACTTCATTGTGAACGCTCTCATATTTTTC------------CTTCCCTTGGCCCCGCGT-CAAGGCGTTT-T--CCCTGCGTAAACGTCACGGAGAGCCACTAGAGTGGAGATGTT
BSTEL_0697                       TGTGTCGCGCGATGTGCGTGTGAGCGGTAACAACGTTGACGTTGCAAGGAAATCGTCAACCA--TCGTTC-GACGCGT------------------CGTCACGTAATGCGAGTACATTGGTAGCCGT
BISA_1443                TAACGTGATGAAAGTCACATATAGATTGTGAGCGCTAACAACCATTGGGA---ATACAAGGTTTTCTGAT--TTTCGT-AGCAAATTTT---TGGGAGCGCAAACGTCTAACCGGGTTTATAAAGTAAAACCGTT
Bbr_1273                 TAACGTGACGAAAATCACATAATGGCTGTGAGCGCTAACAACTGCTGAAA---ACATTAGGGTTATGGCA--TCATTGGATATGCATTG---TGGCCCGCGAAACGTCTTAGCGGGTTTATAAAGTGAAACCGTT
Blon_0840                TAACGTGATAAACATCACAGTTTGTTTGGGAGCGCTAACAACCGTTGAGA---ACATTACCATACCGTTA--TCAAACAGGGTGGTGCG---CTGGTAGCAAAACGTCTTAGCGGGTTTATAGAGTGAAGACGTT
BL1308                   TAACGTGATAAACATCACAGTATATTCGTGAGCGCTAACAACCGTTGAAA---ACATTACCATACGGTTA--TCAAACAGGGTGGTGTG---CTGGTAGCAAAACGTCTTAGCGGGTTTATAGAGTGAAGACGTT
B8809_RS06070            TAACGTGATAAACATCACAGTATATTCGTGAGCGCTAACAACCGTTGAAA---ACATTACCATACGGTTG--TCAAACAGGGTGGTGTG---CCGGTAGCAAAACGTCTTAGCGGGTTTATAGAGTGAAGACGTT
BBNG_00458                       TGCCGCTGACGCAATGTCTGTGAGCGTTCACAACAATGTGACGCAAATCACCTTGCCTTCG---GGGCGA-TAATGACTACGGCAATCAGCGCAAACGTCATCATGTGTAGATACAGTAAGTGTGTT
BIFANG_03283                     TGGTAGACACGTGACCGATGTGAGCGCTGCCAGT----------------------------------------------------------------GTCAGGTTATGTGAGTACTGTTGAAGTGAT
BMERY_0024                       ACGTGGCACGCGGTGATATGTGAGCGCTGCCAGT----------------------------------------------------------------GTCAGGTTATGTGAGTACAGTTGAAGTGAT
THER5_1329                       TTCTTGATACGACTTCATTGTGAACGCTCTCATATTTTTC------------CTTCCCTTGGCCCCGCGT-CAAGGCGTTT-T--CCCTGCGTAAACGTCACGGAGAGCCACTAGAGTGGAGATGTT
BDP_1558                         GTGAGTGCCCGCCATGTTTGTGAGCGCTCACATCAAGTGTGGCGCAAATCACGTTTCAGCCA--TGTCAG-GGCGTGCTAC---ACAAGCCTGAAACGTCATGCTGTGTGGATACAGTAAAAGTGTT
BMOU_0293                        TGGTGGTCCCCACGGTTTTGTGAACGTTCACACCAATTGTGATGCAAATCACGTTTCAGTCA--TGACGG-GCTGTGCTAG---ACGGGTCCGAAACGTCATACTGTGTGGATACAGTAAAAGTGTT
BBPC_1284                        CGCAACGAACACAGCGTTTGTGAGCGCTCACATCTTTTGTGATGCATATCACTTTTCATTAT--TTGCAA-GTCGTGTTAT---AACGCCTTGAAACGTCATGCTGTGTGGATAAAGTAAAGATGAA
BBKW_1423                        CGCAACGGGCACAGCATTTGTGAGCGCTCACATGCTTTGTGAGGCATATCACTTTTCAGTAC--CTGCAA-GTCGTGTTAC---AACGCATCAAAACGTCATGCTGTATGGATAGAGTAAAGATGAA
BSTER_1405                       TTCAACGGGCGCAGTGTATGTGAGCGCTCACAAAAGCTGTGACGAAAATCACTTTTCAGTGG--TTGCAA-ATCATGCGGA---TTCACGGCGAAACGTCATGCTGTATGGATAAAGTAGGGTTGAA
                                                   * ** ** *  **                                                                ***           **   *

BBOU_0652                AСTGAAT-----GAAACCCGTCACAATCGGGTCAT-----TGC------CAAGGAGAAGTAGCAA----ATGGTGAATTCAGCTGTAAAGCCCACGAAGCTCGCTATCATCGGTGCCGGCGCTGTCG
D805_0597                ACTGAAT-----GAAGCCCGTCACAATCGGGTCAT-----TGC------CAAGGAGAAGTAGCAA----ATGGTGAATTCAGCTGTAAAGCCCACGAAGCTCGCTATCATCGGTGCCGGCGCTGTCG
BSTEL_0697               TGGTTG---AATGATGCCCGG---CAACGGGCATT-----GAC----TCT-AAGAGAGGTTCATTAAC-ATGGCTGAATTGGTCAACAAGCCCACGAAGCTCGCTGTCATCGGCGCCGGCGCCGTCG
BISA_1443                AGTTACATGG--CCTGCCATTCT---GCAGACCGC------CT-----TTGAAGAGAGGTTAATCCATCATGGCGGAAACTACCGTTAAGCCCACGAAGCTTGCAATCATCGGTGCTGGTGCTGTTG
Bbr_1273                 AGTTACAAGG--CCTGCCATTCATCAGCAGACCGC------CT-----TTGAAGAGAGGTTTATCCATCATGGCGGAAACTACCGTTAAGCCCACGAAGCTTGCTGTTATTGGTGCCGGTGCCGTTG
Blon_0840                AGTTACAAGG--CCTGCCATTCATCAGCAGACCGC------CT-----TTGAAGAGAGGTTCATCCATCATGGCGGAAACTACCGTTAAGCCCACGAAGCTTGCTGTTATTGGTGCCGGTGCCGTTG
BL1308                   AGTTACAAGG--CCTGCCATTCATCAGCAGACCGC------CT-----TTGAAGAGAGGTTCATCCATCATGGCGGAAACTACCGTTAAGCCCACGAAGCTTGCTGTTATTGGTGCCGGTGCCGTTG
B8809_RS06070            AGTTACAAGG--CCTGCCATTCATCAGCAGACCGC------CT-----TTGAAGAGAGGTTCATCCATCATGGCGGAAACTACCGTTAAGCCCACGAAGCTTGCTGTTATTGGTGCCGGTGCCGTTG
BBNG_00458               GTCAAA-AT---GAACCCCGCCAAACACGGGGCAC-----TGT----GCT-AAGAGAGGTCTAGCAAACATGGTGGATTCACCCATTAAGCCCACAAAGCTCGCCATCGTCGGTGCCGGCGCAGTCG
BIFANG_03283             GTTGTGAGAAATGATGCGCCC---AACCGGCGCAT-----CTC--TC--T-AAGAGAGGTATAAAC---ATGGCAGAATCGCTTATCAAGCCCACGAAGCTTGCTGTTATCGGTGCCGGCGCAGTCG
BMERY_0024               GTTTTGAGGAACGATGCGCCC---AAACGGCGCAT-----CTC--TC--T-AAGAGAGGTATAGCC---ATGGCAGAATCGCTTATCAAGCCCACGAAGCTTGCTGTTATCGGTGCCGGCGCAGTCG
THER5_1329               ACTGAAT-----GAAGCCCGTCACAATCGGGTCAT-----TGC------CAAGGAGAAGTAGCAA----ATGGTGAATTCAGCTGTAAAGCCCACGAAGCTCGCTATCATCGGTGCCGGCGCTGTCG
BDP_1558                 ATTGAAGAT---GAAGCCCGTCACCAACGGGACAT-----TCG--TTGCCAAAGAGAGGTAAGCAC---ATGGTGGATTCACCAATCAAGCCCACAAAGCTCGCTATCATCGGTGCCGGCGCAGTCG
BMOU_0293                ATTGAAGAT---GAAGCCCGTCACCAACGGGACAT-----TCGT-TTGCCAAAGAGAGGTAAGCAC---ATGGTGGATTCACCAATCAAGCCCACAAAGCTCGCTATCATCGGTGCCGGCGCAGTCG
BBPC_1284                ATTCTAAGAG--AAAACCTGCTACCAGCGGGACAT----TCTT-TCTTAT-AAGAGAGGTAAGCAC---ATGGTGGATTCACCAATCAAGCCCACAAAGCTCGCTATCATCGGTGCCGGTGCAGTCG
BBKW_1423                ATCCTAAGGA--GAAACCTGCTACCAGCGGGACAT----TCTTTTCTTGT-AAGAGAGGTAAGCAC---ATGGTGGATTCACCAATCAAGCCCACAAAGCTCGCTATCATCGGTGCCGGTGCAGTCG
BSTER_1405               ATCGAAAGAA--AATACCCTGCCACAACGCGGGACGTTCTTCTT-GCT-CAAAGAGAGGTAAAGCAC--ATGGTGGATTCACCAATCAAGCCCACAAAGCTCGCTATCATCGGTGCCGGCGCAGTCG
                                 *          *                       * **** **        ****   *         ******** ***** **   *  * ** ** ** ** ** *
```

9. *galM* (repression)

```
BCAL_0666       TTGCCTACAAACAGCTCCGCTGTTTGCTTCACGG----------CAACGTCCCATCAGAGGGGA-------------CCAAGGGTGCGGCAGTCCTCGCACTGAGGTGAGGTATCATTGTGAGCGATAACAATAC
Bbr_1237        GTTATGAAGTAGCCCAGAAGTAATCCAGAAATAGCCCTATGAATTGGCTCTCCTCATTGAGGAGAGCCAATTCTGTTATCTATATA-CGCCGCGTCCCAACGCTGATGCGGTACGATTGCGAGCGGTAACAATAC
Blon_0896       GCTACGAAGTAGCCCAGAAGTAGTTCCGTTACGT----------TGGCTCCCCGCCAGAGGGGAGCCAATTATTTATCCTTATGTGCCGCCGCGTCTTGGGACTTGTGCGATACGATTGTGAGCGGTAACAATCT
BL1359          GCTACGAAGTAGTCCAGAAGTAGTTCTGTTACGC----------TGGCTCCCCTCCAGAGGGGAGCCAATTTTTTATCCTTATGTGCCGCCGCGTCTTGGGACTTATGCGATACGATTGTGAGCGATAACAATTT
B8809_RS05795   GCTACGAAGTAGTCCAGAAGTAGTTCTGTTACGC----------TGGCTCCCCTCCAGAGGGGAGCCAATTTTTTATCCTTATGTGCCGCCGCGTCTTGGGACTTATGCGATACGATTGTGAGCGATAACAATTT
                      *   *   *     *  *      *                *   **       **                      *   ** *              ** * **  **** ***** *******

BCAL_0666       G-TCTCATCATCCACGAAGGAGTGTGCTCAAGACCATGGTTCTCAAACCCCGTACCGGCAACCAGTATTCGATTCGCAAGGGCGAGTGGAGCGCCGTCGTCACCGAGCTCGGCGCCAGCCTGCGCGAGCTCAAGT
Bbr_1237        GCTTTCAC-------AAAGGAGTGTGC-----ACTATGGCTTTGAAGCCTCGCACCGGTAACCAATACGCCATCTCATCCGGCGAATGGAGTGCCGTTGTCACTGAGCTGGGCGCCAGCTTGCGCGAACTGAAAT
Blon_0896       G-TTTCAC-------CAAGGAGTGTGC-----ACTATGGCTTTGAAGCCTCGCACTGGTAACCAATATGTCATCTCGGCCGGCGAATGGGGTGCCGTCGTCACCGAAATGGGTGCCAGCCTGCGCGAGCTGAAGT
BL1359          G-TTCCAC-------AAAGGAGTGTGC-----ACTATGGCTTTGAAGCCTCGCACTGGTAACCAATATGTCATCTCGTCCGGCGAATGGAGTGCCGTCGTCACCGAACTGGGTGCCAGCCTGCGCGAGCTGAAGT
B8809_RS05795   G-TTCCAC-------AAAGGAGTGTGC-----ACTATGGCTTTGAAGCCTCGCACTGGTAACCAATATGTCATCTCGTCCGGCGAATGGAGTGCCGTCGTCACCGAACTGGGTGCCAGCCTGCGCGAGCTGAAGT
                * *  **         ***********     ** **** * * ** ** ** ** ** ***** **    **       ***** *** * ***** ***** **  * ** ****** ******* ** ** *
```

10. *pta - ackA* (repression)

```
BSTEL_0445      CTTTCCGCGTTTTT-----------------------CCCGGCAACACGGACGTGAGCGATAACAAGC---GCCGCTACAATAAGGGGTGGTACATCCGCACTGAATCATCAAAGGAGACTGACAC-CAGTGACC
BISA_1342       TCTTCCCGCAATTA-----------------------CTCCGCGTCTTAAGCGTGAACGGTAACAATG---GTCAGTACAATGATGTGTGGTCCTAATTATGTTGATCATCAAAGGAGACAGAC-CAAAGTGACC
Bbr_0772        TCGATGGCAATGCTGAGCCTTGTCTTCGGGCATCCCGCCGCGACACAACACCGTGAACGATAACAACG---ACCACTACAATGAGATATGGTCCCACATATGTTGCACATCAAAGGAGACAGACACA--GTGACC
BL0968          AGCGTCGCAAAGGGGACGC--TTC---------CGATTGGCGCGACACCGGCGTGAGCGATAACAGTAATCGCCACTACAATGAGGTGTGGTCCCTATCGTGTTGATCATCAAAGGAGACAAAAACATCGTGACC
B8809_RS03270   AGCGTCGCCAAGGGGACGCTCTTC---------CGATTGGCGCGACACCGGCGTGAGCGATAACAGTAATCGCCACTACAATGAGGTGTGGTCCCTATCGTGTTGATTATCAAAGGAGACAAAAACATCGTGACC
                                                         *   *     ***** ** *****  *     *  ****** *    **** *        *     ************  *  *   ******
```

11. *pflB - pflA* (activation)

```
                                   AraQ site 1    AraQ site 2     Crp site
BCAL_1553       CCGATTTCACTCTTGGTGTGAGCGATCACTGTGAACGATAACGGAATGTGAGATAGCTCACGCGTATTTTGCGTTAACTCCCAGTGACCAACGGCTAGACTATGGAAGT----AACGACGACAACGGTGCGTCGC
BREU_0248       GTTGGACCGGCCCAAAGGCGTTGGAATGATAACGCTCACAAGTGAATGTGAGATAGCTCACGCATGAATTCAGCCTTTCATAAGAATGTGCAAACTACACTGA-GCATT----AATGGTTCCGTGAAGATGCGAA
BISA_1821       ATGGTCTGCGGCGCAAAACGTTGCAATATCAAGCAAATCACACGAATGTGATATAGCTCACTTGCAATTTCAGCTTTTCCCAAGCATGTGCAAACTACACTTA-GTGGC----AATGGTTCCGTGAAGATGCGAG
Bbr_0787        CGGCGGATGCTGGAAACCGCTGGATTGGTAGCGCTCACAGAA-GAATGTGAGATAGCTCACTCCCAATTTCAGCTTTTCTCAAGACTGTGCAAACTACACTAGTGAAGC----AAGGGTTCCGTGAAGATGCGAA
BL0951          CATCAATACCTAGAAGCCATTGGAATGGTAGCGTTCACAAAAACAATGTGAGATAGCTCACTCCCAATTTCAGCTTTTCTCAAGCAAGTGCCAACTACACTGTTGAACC----AAGGGTTCCGTGAAGATGCGAA
B8809_RS03410   CATCAATATCTAGAAGCCATTGGAATGGTAGCGTTCACAGAAACAATGTGAGATAGCTCACTCCCAATTTCAGCTTTTCTCAAGCAAGTGCCAACTACACTGTTGAACC----AAGGGTTCCGTGAAGATGCGAA
BBSC_1654       TCCTACGACGACGGCCTCGACGGCGATGATGTGAGCGATAACAAAATGTGAGATAGCTCACGTTGTGAATCAGCCTTTTCCCAGCTTGGCTCAACTACCATGGGGAGATGTAAGGAGACGGCGTGAAGATGCGCC
BCHO_1335       TTCACATCATTCGGAAGTGACGCGTATCACAGGGAAAACGGGCGAATGTGACCAAGGTCACGCATCCGCGCGGCCGTTCGCCGATGATGAACGGCTATCATGGGACACG------CAACGGCACAAAGAGGCGTC
BSAE_0970       CGACGGCCCGCCGTTCCGACCGCGGTTATGTGAACGGTAACGAAAATGTGATGTAGCTCACGTCGCGTCCCAGCTTGTTTTCAGGCGGCTGCGGCTACAATGAGGAACC----AACGACGGCGTAACGATGCGTC
BMON_0545       CGTGACGTGAACGTTCACACGATATGGTGCGTATGCCACGGTGCAGTGTGAGGTAACTCACGTTGAGTTCCGAGACGTTGTCGACTAAGGGTGCCTACAATGGCCTCAG----GCGGGGCGCATGAGA--GCGTT
                                      *                     * *****   *  ****                                 ***   *                    *        *

BCAL_1553       AGGAAC-AC-AACACC-AAGGAGTGGATATGACAGCTGTGGAATCTACCGCGCTCACTCAGGAGGAGCTGGACGCCAAGGCGTGGGAC
BREU_0248       ACCG-CAAC-CCCATTAAAGGAGTGAC-ATGACCGCAGTAGAGAATGCCGCTGTCTTCCAGGAGGAGCTCGAGGCCAAGGCGTGGGAT
BISA_1821       ACCAACAAC-CCCACTAAAGGAGTGAC-ATGACCGCAGTAGAGAATGCAGCTGTCTCCCAGGAGGAGCTCGACGCAAAGGCGTGGGCA
Bbr_0787        ACCTACAAC-CCCAT-TAAGGAGTGAC-ATGACCGCAGTAGAAAATGCAGCTGTCTCCCAAGAGGAGCTCGACGCCAAGGCGTGGGCC
BL0951          ACCTACAAC-CCCATTTAAGGAGTGAC-ATGACCGCAGTGGAAAATGCAGCTGTCTCCCAGGAGGAGCTCGACGCCAAGGCGTGGGCC
B8809_RS03410   ACCTACAAC-CCCATTTAAGGAGTGAC-ATGACCGCAGTGGAAAATGCAGCTGTCTCCCAGGAGGAGCTCGACGCCAAGGCGTGGGCC
BBSC_1654       GCCTACGAC----ACC-AAGGAGTGAC-ATGACAGCAGTTGACAATGCAG---TGTCTCAGGAAGAGCTCGAGGCCAAGGCCTGGGCA
BCHO_1335       GCAACA-GA-CCCCAA-AAGGAGTGAATATGGCAGCAGTCGAGACAACCGCGGTCTCCCCGGAGGAAATCAAGGAGCAGGCCTGGAAG
BSAE_0970       GCCGACGATCACCCAG-AAGGAGTGGATATGACAGCAGTTGATGCAACCGCGGTCTCCCCGGAGGAGCTCAACGCCAAGGCGTGGGAC
BMON_0545       TCGTACAAC----ACG-AAGGAGTGATTATGACAGCAGTCGAAGAAGCCGCTCTGTCTCCCGAGGAACTCAAAGCACAAGCCTGGCAT
                                 ********   *** * ** ** **     * *   *    *  ** **  *  * *   * ** ***
```

12. BL1113 (repression)

```
BCAL_0028       TGCAACACGCGCGGTGGCG----CGGTGTGGGTTTGCGTTCAAGTGTGGTTGAGGTGTTTCCAT-GGACGTGTTAGCGTTCACGGCC----GGTTGCGATGGAGATACCCGGATCGCCGGCGGAACCAGGAACCA
BREU_0675       TGCGACACGCGAGGTGACG----CGGTGCGGGTTTGCGTTCAAGTGCGGTTGAGGTGTTTCCAT-GGACGTGTTAGCGTTCACGGCC----GGTTGCGACGGAGACACCCGATCGGCGCGTGGAACGCGGAACCG
BISA_0103       -----CACGCGCGACCAAC----TCATGTCAGCTTGCGTTCAAGTGTGGTTGAACTGTTTCAATATGAATTGTTAGCGCTCACGGAGACACCGTGATATTATCGATAAATGGCATGTGCGCGTATGGAGCCAC-
B8809_RS02480   AGTCACCACCGGCGTGTCGCAATAGCCGCCAACTTGCGTTCAAGTGGGGTTGAACTGTTTCAATAAGAATTGTTAGCGTTCACGGCC----GG------------------------------------------
                     *   **                *     ************* ******  ****** **  **  ******** ******     * *

BCAL_0028       TGTGAGCCGTACCAGTGCTGCAACGGATTGCAGCGGAAGGAGCATCAATGAAGTACCGCAATGTAGGCAAGTCCGGACTGAAGGTCAGCGAGGTCGCACTGGGAAGCTGGGTCACCCAGCTGACGAACGACGCGC
BREU_0675       CAGGAGC---------------------TGCAAACGAAGGAGCATCAATGAAGTACCGCAGCGTGGGCAAGTCCGGACTAAAGATCAGCGAGATCGCACTGGGAAGCTGGGTGACGCAGCTGACGAACGACGCGC
BISA_0103       ----CGCAAACCACACCGCGCAAGTACTCAACAACGAAGGAGCAACAATGAAGTATCGCAGCGTTGGCAAGTCCGGTCTGAAAATCAGTGAGGTCGCACTGGGCAGCTGGGTCACCGACCTCAAGGGCACCGCCG
B8809_RS02480   ----CCATGAAGAGACGACGGTGACGATTGATTATCAAGGAGCACACATGAAGTATCGCAGCGTTGGCAAGTCGGGACTGAAGATCAGCGAGGTCGCACTGGGCAGCTGGGTCACGGATCTCAAGGGCACGGCGG
                                                    ********   ******** ****  ** ******** ** ** **  **** *** ********** ******** **  * ** * *  *   **
```

13. *malE* (activation), MalR represses the *malE* gene by binding to tandem sites (forms a tetramer)

```
                    AraQ site          MalR site 1                                                                                                 MalR site 2
Blon_2444       ATATGTTAGCGCTCTCATGATTGCGAACGCTTGCATTGTGATAATGAGGCAAAAAGG-CTGAAGTAATAGGATATCTGTTGACAATGGCCTGAAAATGATGATTCATGACGCGCCGCTATTGCTATCGTTTGCAA
BL0141          ATATGTTAGCGCTCTCATAATTGCGAACGCTTGCATTGCGATAATGAGGCAAAAATGACCGAATTAATGGGATATTTGTTGACAATGGCCTGAAAATGATGATTTATGACGCGCCGATATTGCTATCGTTTGCAA
B8809_RS09430   ATATGTTAGCGCTCTCATGATTGCGAACGCTTGCATTGCGATAATGAGGCAAAAATGACTGAATTAATGGGATATTTGTTGACAATGGCCTGAAAATGATGATTTATGACGCGCTGATATTGCTATCGTTTGCAA
                ****************** ****************** *************** * * *** **** ****** ************************************ * ****************

Blon_2444       AAAGAAGCAAACCGTTGCATAATAGTGCATGTCGATAGTCGTAAAGGTGCGTGCTAGCGTACCTGTCCCGACCGGGGATGGCCGGGATCGACGATGAAAGAGAAAGGTTCGATGAT
BL0141          AAAGAAGCAAACCGTTGCACAATAGTGCATGTCGATAGTCATAAAGGTGCGTGTTAGCGTACCTGTCCTGACCGGGGACGGCCGGGGTCGACGATGAAAGAGAAAGGTTCGATGAT
B8809_RS09430   AAAGAAGCAAACCGTTGCACAATAGTGCATGTCGATAGTCGTAAAGGTGCGTGCTAGCGTACCTGTCCTGACCGGGGACGGCCGGG-TCGACGATGAAAGAGAAAGGTTCGATGAT
                ****************** ******************* *********** ************* ******** ******* ****************************
```

14. *malQ1* (activation)

```
Bbr_1650        ----------------------------TAGATTCCGTTTTGCCGCCATGTGAGCGTTCTCATGTCGATATATGGTTGCTCAATTGTTGCAAACGTAAGCATTCTATGGC--CGTCTTACGGTGTGGCGCGGCTT
Blon_2246       TGTGTTTTGCCGCGCAAATTTGGCCGGCAATCACCCCATATCCACCATTGTGAGCGCTCACATCTCACTGTGTGAGGGCTAAAAGTACATAATTTGGCGGAATCACGGCATTTTGCTTACGGTGTGGCGCAATCA
BL1570          TGTGTTTTGCCGCGCAAATTTGGCCGGCAATCACCCCCTGTCCACCATTGTGAGCGCTCACATCTCACTATGTGAGGGTAAAAAGTACATAATTTGGCGGAATCATGGCATTTTTCTTACGGTGTGGCGCAATCA
B8809_RS08220   TGTGTTTTGCCGCGCAAATTTGGCCGGCAATCACCCCCTATCCACCATTGTGAGCGCTCACATCTCACTATGTGAGGGTAAAAAGTACATAATTTGGCGGAATCATGGCATTTTTCTTACGGTGTGGCGCAATCA
                ** **    *    *             ** *    *** * * *  *  ******** ** *** **  * * **   *    **        **       * * **   *        **************

Bbr_1650        TTCGGCGCTACACTGGGGCTATGAGTGAGAATACCGAGAGTAAAGAACGATTGGCTCGTCCGCTGATTCGATTGG
Blon_2246       AACGGCGTTAGACTGGGGCTATGAGTGAGATTACCGAGAGTGAAGAGCGTCTGGCCCGTCCGTTGATTCGACTGG
BL1570          AACGGCGTTAGACTGGGGCTATGAGTGAGATTACCGAGAGTGAAGAGCGTCTGGCCCGTCCGTTGATTCGACTGG
B8809_RS08220   AACGGCGTTAGACTGGGGCTATGAGTGAGATTACCGAGAGTGAAGAGCGTCTGGCCCGTCCGTTGATTCGACTGG
                  ***** ** ****************** ********** **** **  **** ****** ******** ***
```

15. *maa* (repression)

```
BDP_0569        ACCTCTGCCGCCGATTCGCCAATTT-GTTAGATGGTCGAAATAATATGTTGCGTGAACGCTCACAACATACGCGGGAAA--TGGAAGACGGGAGA-GGATATGGCACG
BMOU_1838       ATCGTCCTGCCCGATTCGCCAATTT-GTTAGATGGTCGAAATAACATATTGCGTGGGCGTTCACAGCA--TAAACGGAAAGCGGAAGGTGGGAGA---GTATGGCACG
BBPC_0474       ACATCGGATAGTATGTCGTATGCCA-GGTCAAGTAGATGAATATAGTATAGTGTGAGCGTTCACTATAGCGTGGTGGAAATCGTGACGAAGGAGACGGGCATGACGAA
BBKW_0464       ACATCGGATAGTATGTCGTATGCCA-GGTCAAGTAGATGAATATAGTATAGTGTGAGCGCTCACTATAGCGTGGTGAAAATCGTGACGAAGGAGACGGGCATGACGAA
BSTER_0445      ATGCCGGGTAACGTTTCATGCGTCATGGTCAAGTAAGTGAATATAGTATGGTGTGAGCGTTCACTATAGTGCGGTGGAAT--GTGATGAAGGAGACGGATATGACGCA
                *              **         * *  *       ****   * * * ***  ** ****   *       * **      *    *****     *** *
```

16. *glgB* - Blon_1762 - Blon_1763 (repression)

```
BREU_1083       CGTGGATAAACGACCATATCGCCGACTTTATAAGACATGAATCCTCCGAGAACTGACGGCTCACACACAAAAACTCGAATAATTGTGCCATCTGGCATGGACTG-CGACGCTGATAGCGTTCACAATATGTACGA
BISA_1317       CGTGGATAGACGACCATATCGCCGACTTGATAAGACATGAATCCTCCGAGAACTGACGGCTCACACACAAAAACTCGAATAATTGTGCCACTTGGCATGGACCGGCCCCAATGAGAACGCTCACATTACCTAAGG
Bbr_0746        CGTGGGTAGACGACCATATCGCCGACTTGATAAGACATGTATCCTCCGAGAACTGACGGATTACTCACAAAAACTCGAATAATTGTGCCATATGGTCTGGACTG-GGACAATGAGAGCGCTCACAATCAAGCGAC
Blon_1761       CGCGGATAGACGACCATATCGCCGACTTTGTAAGACATGTACCCTCCGAGAACTGACGGCTTACACACAAAAACTCGAATAATTATGCCATGCGGTCTGGACCG-CCCCTATGAGAGCGCTCACAACCACGAAGA
B8809_RS03065   CGCGGATAGACGACCATATCGCCGACTTTGTAAGACATGTACCCTCCGAGAACTGACGGCTTACACACGAAAACTCGAATAATTATGCCATGCGGTCTGGACCA-CCCCTATGAGAGCGCTCACAACCACGGAAA
                ** ** ** *******************  ********* * ***************** * ** *** *************** *****    **   *****       *   *** * ** *****

BREU_1083       CACGCGAGCAACAGCAGAATTTCGCCGCATTTGCCGCGTTAATACGGCGTTTTACGCGCTGACACACGTAGACTTTGGGGCATGGATACTGAAACCAAGATCAACGAAGATACCATCACCGTGCCTGTCGCCGAA
BISA_1317       CAGGACAAATTTGACAGAAATTCGATATATTTACCGCGTTAATACGGCGTTTTCAACTTCTACACACGTAGACTTTGGGGCATGGATACTGAAAAGCAGATCAAAGAAGATACCATCACCGTGCCTGTTGCTCAG
Bbr_0746        AACACTCTAAGCGGCATAAATTCGCCATTTTCGACGCGCCAATGTAGCGTTTCCGACACCAACGCACGTAGACTTTGGGGCATGGCTAATGAAACGAAGATCAAGGAAGATACCGTCACCGTGCCTGTTGCCCAA
Blon_1761       CACACCGAAAAGGGCAAGAATTCGCCATTTTCGACGCGCCAATGTAGCGTTTTCGACTCCAACGCACGTAAACTTTGGGTCATGGCTAAAGAAACGAAGATCAAGGAAGATACCGTCACCGTGCCTGTCGCCCAA
B8809_RS03065   CACAACGAAAAGGGCAAGAATTCGCCATTTTCAACGCGCCAATGTAGCGTTTTCGACTTCAACGCACGTAGACTTTGGGACATGGCTAAAGAAACGAAGATCAAGGAAGATACCGTCACCGTGCCTGTCGCCCAA
                 *            **  * ****      **    ****  ***    ******     *     ** ****** ******** ***** **  ****    ******* ********* ************* **  *
```

17. *carD* - *ispF* (repression?)

```
BREU_1084       TTCTGCTGTTGCTCGCGTGTCGTACATATTGTGAACGCTATCAGCGTCGC-AGTCCATGCCAGATGGCACAATTATTCGAGTTTTTGTGTGTGAGCCGTCAGTTCTCGGAGGATTCATGTCTTATAAAGTCGGCG
BISA_1318       TTCTGTCAAATTTGTCCTGCCTTAGGTAATGTGAGCGTTCTCATTGGGGCCGGTCCATGCCAAGTGGCACAATTATTCGAGTTTTTGTGTGTGAGCCGTCAGTTCTCGGAGGATTCATGTCTTATCAAGTCGGCG
Blon_1760       TCTTGCCCTTTTCGGTGTGTCTTCGTGGTTGTGAGCGCTCTCATAGGGGC-GGTCCAGACCGCATGGCATAATTATTCGAGTTTTTGTGTGTAAGCCGTCAGTTCTCGGAGGGTACATGTCTTACAAAGTCGGCG
BL0998          TCTTGCCCTTTTCGTTGTGTTTCCGTGGTTGTGAGCGCTCTCATAGGGGT-GGTCCAGACCGCATGGCATAATCATTCGAGTTTTCGTGTGTAAGCCGTCAGTTCTCGGAGGGTACATGTCTTACAAAGTCGGCG
B8809_RS03070   TCTTGCCCTTTTCGTTGTGTTTCCGTGGTTGTGAGCGCTCTCATAGGGGT-GGTCCAGACCGCATGGCATAATTATTCGAGTTTTCGTGTGTAAGCCGTCAGTTCTCGGAGGGTACATGTCTTACAAAGTCGGCG
                *  **            **          ***** ** * ***   *  *    *****  **    ***** *** *********** ****** ****************** * *********  *********
```

18. *birA* (repression)

```
BISA_1131       GGATGGATATGG-----------ATGGTGGGAGCCTTCTAGGGTGTGTGCGAGCGGCTGTGACTTTGCTCACCTGTGAACGTTCACATGGTCGCGGTATGTAGAAAAGCGGATTTTTGGGTTTGGAACCGTGAGA
Bbr_1723        ATTGCAGACGCAGGCAAGGCCGCTATGACTGCAATCGTATGCGGGCCATCTGCATGGTGTGACTTTGCTCACGTGTGAGCGTTCACACCGAGGTATTGGCCGAAAATGGAGACAT-CGGGAATCTGGTCGCGCAA
Blon_2288       ATTGCCATTGTTCGCAAGCCAATACGACGGCCGGGTGCATGCGTGGGTTCGGCGAATGCGGAATTTTCGAAGTTGTGAACGTTAACACGGGCTATGTGGGTGAAAGTGGGAATATCGCGGGTAAAAAACGGCGCA
B8809_RS08755   ATCGCCATTGTTCGCAAGCCAATATGACGGCCGGGTGCATGCGTGGGGTCGGCGAATGCGGAATTTCCGAAGTTGTGAACGTTAACATGGGCTATGTGGGTGAAAATAGGAATAACGCGGGCATATCGCGCCAAA
                                                        * * *     *           ** *** *  *  ***** **** ***  *       *       **       *       **         **    *

BISA_1131       AATGCGGTAACGTTTTGCCCATGATGCTTGCACTACAGACCACGACGCCT
Bbr_1723        ATCGGGGTAACGTAGTTCCCATGATGATTGCTTTGCAAGCTGCAACGCCG
Blon_2288       ACCGAGGTAACGTAGTGTCCATGATGATTGCATTGAAAGCTGCAACGCCG
B8809_RS08755   ATCGAGGTAACGTAGTGCCCATGATGATTGCGTTGAAAGCTGCAACGCCG
                *  * ********  *  ******** ****  *   *   *   * *****
```

19. *sixA* (repression)

```
BISA_0671       TGGATGCATGCCGATGAATATGTGAACGCTCACAACGGGCTATACTGGAATGACT--------TTGTGTTGTTCGTTGTGAGCTGTGCAAAACCTTGAAG-AGAGGGAGCCTCGAAGTGGGCATCAAGCTGAACA
BREU_0084       CGAGGCTGCCTGAACGATTATGTGAACGCTCACATCAAGCGTATAAT--AGTTTT--------TCTCAATGTTTGTTGTGAGCTACGCATATCC----GG-AAGGGGAGCATAACAATGGGCATCAAGCTGAGCA
Blon_1456       TAACCGCCAAACGATGAGTTTGTGAGCGCTCACACACCTCTATACTGGAAGCAAC--------GCCACACCGCCACACCGGGCAGG-------C-GGTTCGTGAAGGGAGCCACGGATGGGCATCAAGCTGAGCA
B8809_RS04185   TAACCGCCAAACGATGAGTTTGTGAACGCTCACACACCTCTATACTGGAAGCAATACCACACCGCCACACCGCCACACCGGGCAGA-------C-GGTTCGTGAAGGGAGCCACGGATGGGCATCAAGCTGAGTA
                                ** * ***** ********     *     *   *                             * **           *           **          ***************  *
```

20. *glk* (activation)

```
BISA_1968       TAGAACGATTGACGCGATGGAGCTGCGAATGCCAACGTTTGCGCTAACAATCCTTGTTTTGATTGGTTATGGCATGACAACGATTGCTGTGCAAAATATGTTCCGCGGTGAACAAGTTATGTACCTAGTGGTAAT
BREU_2338       -GCCGTCTTGCGAGCGGTTGGGAAACGAATGCTAACGTTTGCGCTAACAATCGTTGTTTTGCAAGGAAGCCGCTGATGGTGATGTATGAGACAGTATATGCCGCCAATCGTGCAGCTTATGTACCTGATGGTAGT
B8809_RS07595   TAGTCTGAATGACAACGTTTGCTATTGAATGACAACGTTAGCGTTAACAGTGCTTATAAATGGTGAAAAACGTGTGCAGGGTCACATTGGCAGAACGTGCGCGAGATATGACAAATCATGAAAAATACGCTATGG
BL1691          TAGTCTGAATGACAACGTTTGCTATTGAATGACAACGTTAGCGTTAACAGTGCTTATAAATGGTGAAAAACGTGTGCAGGGTCACATTGGCAGAACGTGCGCGAGATATGACAAATCATGAAAAATACGCTATGG
                                  *        *****  ****** *** ***** *  ** *       *      *                                      *   *         *  *

BISA_1968       TAAGTCATCACGTTTCGTTATCATCTCAAGTATCTCAACTGATATCTCATAC-TCAAGGAGCGAGTCATGACCAC
BREU_2338       TGGGTCACCCCTCTTCGTTACTATTTCA-GTAGTTCCATAGGAATCCCATAC-TCAAGGAGCGAGTCATGACTAC
B8809_RS07595   GAGGTCATGGCAACTCGTTACTATTTTCGCTGTCTCGTACGCCACACAAGCCTTCAAGGAGTAAGCCATGACCAC
BL1691          GAGGTCATGGCAACTCGTTACTATTTTCGCTGTCTCGTACGTCACACAAGCCTTCAAGGAGTAAGCCATGACCAC
                  ****   *   ******  ** *    *   **    *  *     *  ********  ** ****** **
```

21. Fructosides utilization operon (activation)

```
                                                                                          AraQ site          BfrR site 1          BfrR site 2
BIFANG_03432   AATTGAGGGCGGAAATGGCGGG-GCGGTGCCGATGCCTACGTTTGCATGATGTGGCGGAAATGACGTTATGAGAGCGCTAACAGTTGGAATAATCGTTTAACGTTTGACATTAAACGATTAACGATTTACGGTGTT
BMERY_1312     AATTGAGGGCGGGAATGGCGGGGGTGGTGCCGATGCCTACGTTTGCATGATGTGGCGGAAATGACGTTATGAGAGCGCTAACAGTTGGAATAATCGTTTAACGTTTGACATTAAACGATTAACGATTTACGGTGTT
BAD_1330       GGTGTTCTGCAAGAACGGCAGT-ATGGAGCCGAAGTTTACGTTTGC--GACGCTGGGGGGACTGGTTGATGAGAGCGCTAACTATTGAGATAAACGATTAACGTTTGACATTAAACGATTAACGATTTATAGTGTT
BSTER_1333     GGTGTTCTGCAAGAACGGCAGT-ATGGAGCCGAAGTTTACGTTTGCGACGCTGGGGGGACTGGT--CGATGAGAGCGTTAACTATTGAGATAAACGATTAACGTTTGACATTAAACGATTAACGATTTATAGTGTT
cons             *     **    ** *** *     ** ***** *  *********        * **          ********* ****   ***   **** ** ********************************   *****

BIFANG_03432   AACCACAGCAAGAGCACAACGCACGTGATCTTGAGGTTCGCGTCGGGGCGTACCCGAT------GCGGGAGGAATATTAAGGAAGAAGGAAT---ATCATGGCACGAAGGAGTGTACGCCTGATCGCAGCC
BMERY_1312     AACCACAACAAGAGCACAACGCACGTGATCTTGAGGTTCGCGTCGGGGCGTACCCGAT------GCGGGAGGAATATTAAGGAAGAAGGAAC---ATCATGGCACGAAGGAGTGTACGCCTGATCGCAGCT
BAD_1330       AATCAC--------CGCAAAGCAAGCGGCTGATGAGGTCGTACCGGTGCGTCTTACATCGGTACGAGGAGGAAACATCAAGGAAGAAGGAGCCTAATCATGGCACGAAGAAGTGTTCGCCTGTTGGCATCA
BSTER_1333     AACCAC--------CGCAAAGCAAGCGGCTGATGAGGTCGTACCGGTGCGTCTTACATCGGTACGAGGAGGAAACATCAAGGAAGAAGGAGCCTAATCATGGCACGAAGAAGTGTTCGCCTGTTGGCATCA
cons           ** ***        * *** *** * *         * ***   *** ****     **       * **  * ** ** ************     ************** ***** ****** * *** *
```
